# A Unique Modification of the Eukaryotic Initiation Factor 5A Shows the Presence of the Complete Hypusine Pathway in *Leishmania donovani*

**DOI:** 10.1371/journal.pone.0033138

**Published:** 2012-03-16

**Authors:** Bhavna Chawla, Ravi Ranjan Kumar, Nidhi Tyagi, Gowri Subramanian, N. Srinivasan, Myung Hee Park, Rentala Madhubala

**Affiliations:** 1 School of Life Sciences, Jawaharlal Nehru University, New Delhi, India; 2 Molecular Biophysics Unit, Indian Institute of Science, Bangalore, India; 3 Oral and Pharyngeal Cancer Branch, National Institute of Dental and Craniofacial Research (NIDCR), National Institute of Health, Bethesda, Maryland, United States of America; Technion-Israel Institute of Technology, Israel

## Abstract

Deoxyhypusine hydroxylase (DOHH) catalyzes the final step in the post-translational synthesis of an unusual amino acid hypusine (*N* ^€^-(4-amino-2-hydroxybutyl) lysine), which is present on only one cellular protein, eukaryotic initiation factor 5A (eIF5A). We present here the molecular and structural basis of the function of DOHH from the protozoan parasite, *Leishmania donovani,* which causes visceral leishmaniasis. The *L. donovani* DOHH gene is 981 bp and encodes a putative polypeptide of 326 amino acids. DOHH is a HEAT-repeat protein with eight tandem repeats of α-helical pairs. Four conserved histidine-glutamate sequences have been identified that may act as metal coordination sites. A ∼42 kDa recombinant protein with a His-tag was obtained by heterologous expression of DOHH in *Escherichia coli*. Purified recombinant DOHH effectively catalyzed the hydroxylation of the intermediate, eIF5A-deoxyhypusine (eIF5A-Dhp), *in vitro*. *L. donovani* DOHH (LdDOHH) showed ∼40.6% sequence identity with its human homolog. The alignment of *L. donovani* DOHH with the human homolog shows that there are two significant insertions in the former, corresponding to the alignment positions 159-162 (four amino acid residues) and 174-183 (ten amino acid residues) which are present in the variable loop connecting the N- and C-terminal halves of the protein, the latter being present near the substrate binding site. Deletion of the ten-amino-acid-long insertion decreased LdDOHH activity to 14% of the wild type recombinant LdDOHH. Metal chelators like ciclopirox olamine (CPX) and mimosine significantly inhibited the growth of *L. donovani* and DOHH activity *in vitro*. These inhibitors were more effective against the parasite enzyme than the human enzyme. This report, for the first time, confirms the presence of a complete hypusine pathway in a kinetoplastid unlike eubacteria and archaea. The structural differences between the *L. donovani* DOHH and the human homolog may be exploited for structure based design of selective inhibitors against the parasite.

## Introduction

Hypusine (*N* ^€^-(4-amino-2-hydroxybutyl) lysine) is a unique amino acid present in the eukaryotic initiation factor 5A (eIF5A) [1]. Hypusination of eIF5A involves spermidine dependent biosynthesis of hypusine [2] on one specific lysine residue of eIF5A. Hypusine formation occurs exclusively on eIF5A and is necessary for its biological roles in cell growth and survival. It is synthesized in two enzymatic steps [3]. The first step is catalyzed by the enzyme deoxyhypusine synthase (DHS) [EC 2.5.1.46] which catalyses the NAD^+^ dependent transfer of the 4-aminobutyl moiety of spermidine to a specific lysine residue of the eIF5A precursor protein to form an intermediate, deoxyhypusine [4], [5]. This intermediate is subsequently hydroxylated by the enzyme deoxyhypusine hydroxylase (DOHH) [EC 1.14.99.29] which completes the synthesis of hypusine and maturation of eIF5A [6]. Disruption of the eIF5A and DHS genes has been found to be lethal to *S. cerevisiae* [7], [8]. 

The first step in the modification of lysine to deoxyhypusine is reported to occur in all archaea. However, no orthologs of the second enzyme, DOHH have yet been reported in the archaeal genomes or proteomes [9]. Interestingly, despite the lack of evidence for DOHH, archaeal species have been found to contain either hypusine or deoxyhypusine or both [10]. On the contrary, there is no evidence for the occurrence of deoxyhypusine or hypusine in eubacteria. However, phylogenetic analysis showed the presence of DHS-cognate genes in several bacterial species [11].

The DOHH gene is found to be essential in *C. elegans* [12] and *D. melanogaster* [13] but not in *S. cerevisiae* [14]. DOHH seems to be functionally more significant in the yeast, *S. pombe,* in comparison to *S. cerevisiae,* where a mutation in the gene caused a temperature sensitive growth and abnormal distribution and morphology of mitochondria [15]. The protein DOHH has only been recently identified and characterized [14], [16], [17]. Unlike DHS, its catalytic properties are not very well understood. Sequence analysis reveals that DOHH belongs to a family of HEAT-repeat containing proteins (which includes Huntingtin, Elongation Factor 3, a subunit of Protein phosphatase 2A and target of rapamycin) and consists of eight tandem HEAT-repeats organized in a symmetrical dyad [14]. It is a metalloenzyme and requires a di-iron active center for its activity [18]. It also contains four strictly conserved His-Glu motifs which are essential for binding iron and catalysis [16]. Like other protein hydroxylases, DOHH is inhibited by various metal chelators, for example mimosine, 2, 2′-dipyridyl, deferoxamine and ciclopirox (CPX). These metal chelators inhibit HIV-1 multiplication and gene expression by inhibiting DOHH and therefore, DOHH has been suggested as a potential target for anti-retroviral therapy [19], [20].

*Leishmania donovani* is a protozoan parasite and is the causative agent of visceral leishmaniasis. The parasite life cycle consists of two morphologically distinct stages. The promastigote forms live inside the gut of the sandfly and the amastigote forms reside in the macrophages of the mammalian host. The control strategy relies mainly on chemotherapy. The existing repertoire of drugs is limited. With the growing incidence of resistance to the existing drugs, there is a pressing need to look for newer drugs and drug targets. In view of the essential nature of hypusine in eukaryotic cell growth and survival, the hypusine pathway presents a potential new target for anti-parasitic therapy. We have recently reported two *DHS-like* genes in *L. donovani* which show low homology with the human DHS [21]. Both genes were cloned and expressed, but only one, *DHS34*, exhibited deoxyhypusine synthase activity. Gene replacement studies for *DHS34* indicated that the enzyme deoxyhypusine synthase and eIF5A modification play an essential role in cell viability of this pathogenic organism [21]. Furthermore, we also reported that the inhibitors known for this pathway in humans are not effective against *Leishmania* [21]. Despite conservation of some of the active site amino acid residues between the human and leishmanial DHS, a potent inhibitor of human DHS, *N* ^1^-guanyl-1, 7-diaminoheptane, had little inhibitory effect on either *L. donovani* proliferation or recombinant DHS34. This finding suggests a topological difference in the spermidine binding sites between the human and the leishmanial enzymes and opens the possibility that the differences between the two enzymes could be exploited for drug development for visceral leishmaniasis.

This study combined with our previous studies, reveals that the complete hypusine biosynthetic pathway is present in *Leishmania*. We report for the first time the presence of this pathway in a kinetoplastid. To understand the hypusine biosynthetic pathway of *L. donovani*, we have cloned and characterized the second enzyme, deoxyhypusine hydroxylase (DOHH), which completes the synthesis of hypusine and maturation of eIF5A. Sequence analysis of *L. donovani* DOHH indicates that it is highly α-helical and has 40.6% sequence identity with the human homolog. Metal chelators like CPX and mimosine significantly inhibited the growth of *L. donovani* and also the activity of recombinant DOHH *in vitro*. These inhibitors were much more effective against the *L. donovani* than the human enzyme. Alignment of the *L. donovani* DOHH sequence with the human homolog showed two insertions in the former and one of the insertions was found to be crucial for its activity. Superposition of the modeled structures of human and *L. donovani* DOHH showed differences in the C-terminal His-Glu motifs. The structural differences between the *L. donovani* DOHH and the human homolog might account for the differences in the inhibitor binding properties of the parasite compared to those of the human homolog.

## Results

### Sequence Analysis and Genomic Organization

Sequence analysis, database search, and alignment of the *L. donovani* DOHH amino acid sequence were performed as described in Materials and Methods. The LdDOHH amino acid sequence had a single open reading frame consisting of 981-bp (*L. donovani DOHH*, HM138676). The ORF encoded a putative polypeptide of 326 amino acids, with a predicted molecular mass of ∼36 kDa. Amino acid sequence alignment of the DOHH protein with homologous sequences from other species revealed that it shares 99.4% identity with *L. infantum* (LinJ26_V3.1920), 95.1% identity with *L. major* (LmjF26.1910), 61.1% identity with *T. cruzi* (Tc00.1047053507615.70), 61.2% identity with *T. brucei* (Tb09.160.1240), 40.6% with *H. sapiens* (NP_112594) and 36.2% with *S. cerevisiae* (P47120) proteins.

It was reported earlier that the human DOHH protein sequence contains eight HEAT-repeat domains [16]. Sequence analysis of the *L. donovani* DOHH protein showed the presence of eight tandem HEAT repeats. It also showed the presence of the four conserved His-Glu motifs, which are conserved in all eukaryotic homologs (Figure S1). These conserved histidine and glutamic acid residues have been reported to be absolutely necessary for catalysis and iron binding [16]. A phylogenetic tree has been generated (Figure S2) for the putative DOHH sequence with the other homologous sequences. The tree indicates a closer evolutionary relationship of *L. donovani* DOHH with *Trypanosoma brucei* (among the kinetoplastid protozoa) and other eukaryotic pathogen species such as *Plasmodium falciparum*. 

The *Leishmania* DOHH protein sequence shares ∼16% and 40.6% sequence identity with *E. coli* hypothetical protein YibA and human DOHH (hDOHH) respectively. As depicted for hDOHH, the *Leishmania* protein sequence also has eight HEAT repeats. Repeats 1–4 are present in the N-terminal domain and repeats 5–8 are present in the C-terminal domain of the protein [14].

**Figure 1:**
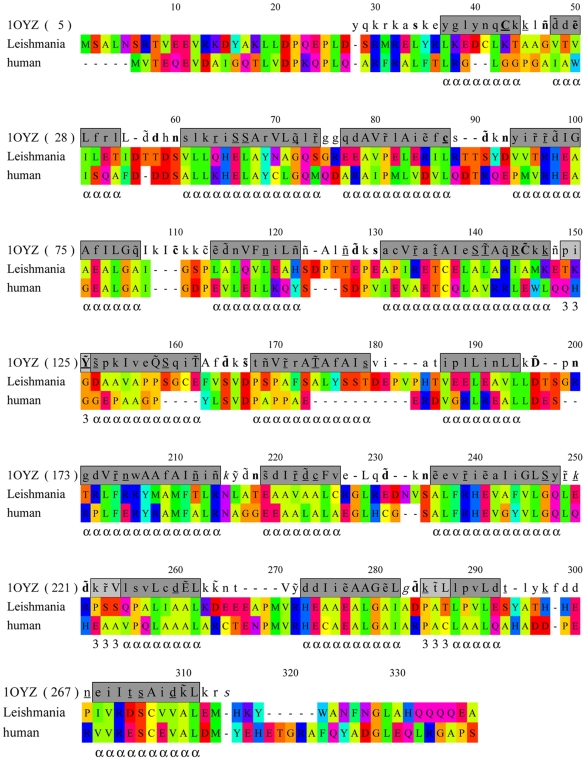
Comparison of the alignment and structural features of human (NP_112594) and *L. donovani* DOHH (ADJ39999) protein sequences with respect to the hypothetical protein YibA (NP_290174) of *Escherichia coli*. This alignment gives an indication of structural features which would be expected for the *L. donovani* and human DOHH protein sequences. Residues in human and *L. donovani* DOHH are color-coded according to the chemical property of the amino acids. The number in parentheses after the protein code indicates the PDB residue number at the beginning of each block. The top line shows alignment positions. The gray block corresponds to residues in alpha helices. Key to the formatted Joy alignment of the *E. coli* DOHH (1OYZ) structure: Solvent inaccessible residues –UPPERCASE; Solvent accessible residues – lowercase; positive ϕ – *italics; cis*-peptide – breve ă; hydrogen bond to the other side chain – tilde ỹ; hydrogen bond to the main chain amide – bold; hydrogen bond to the main chain carbonyl – underlined; disulfide bond – cedilla ş.

**Figure 2:**
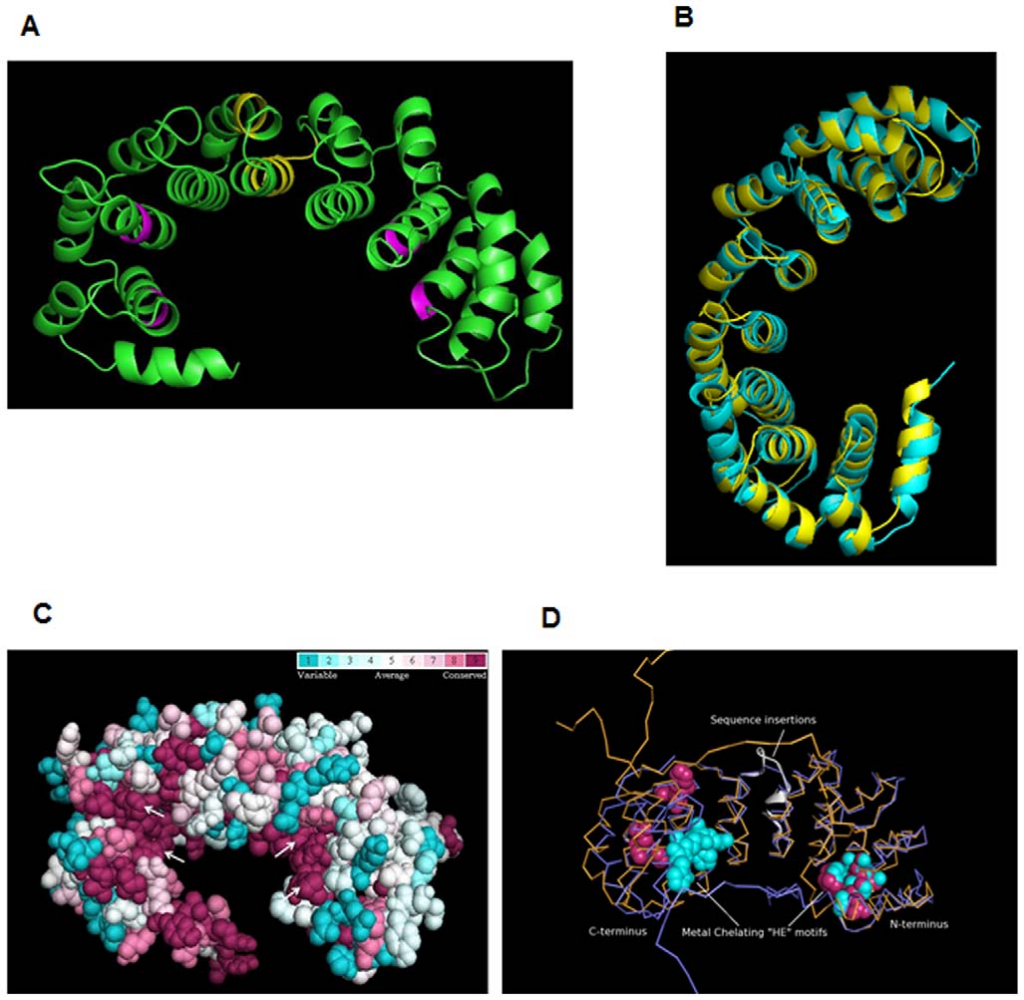
Structure Modeling of DOHH. (A) Model of the *L. donovani* DOHH protein generated by using the *E. coli* hypothetical protein YibA (Protein data bank accession ID: 1OYZ) as a template. As expected from the template structure, the generated model is rich in α-helices. The conserved His-Glu motifs are colored pink and are present at the inner concave surface of a horseshoe shaped structure. The amino acid insertions are colored yellow and are present at the outer surface and the inner concave surface of the generated model. (B) Superimposition of the *L. donovani* DOHH model (cyan) and the *E. coli* hypothetical protein YibA (yellow). The generated model and the E. coli structure superimpose with an RMSD of 1.6 Å. (C) Conservation pattern obtained for generated model of DOHH protein of *L. donovani*. DOHH is represented as a space-filled model and colored according to the conservation score. Fully conserved His-Glu motifs are marked by white arrows. The coloring scheme is depicted in the color-coding bar. All four His-Glu motifs are also represented. As is quite evident from the figure, the amino acid residues lining the inner concave surface are quite conserved as compared to the amino acid residues present on the outer surface. (D) Structural superposition human DOHH (violet) with that of *L. donovani* DOHH (Orange) is shown as a Ribbon diagram. The RMSD of the structural superposition is 4.8 Å. Metal chelating “HE” motifs from human (Pink) and *L. donovani* (Cyan) are shown as spheres. The sequence insertions in the *L. donovani* DOHH (white) are shown as cartoons.

The secondary structural features of human and *L. donovani* DOHH protein sequences with respect to *E. coli* YibA protein are represented in Figure 1. YibA is a predicted bacterial lyase with HEAT-repeat structures and is not expected to possess deoxyhypusine hydroxylase activity due to lack of active site residues. YibA was selected from pre-existing crystal structure data base as a template to model leishmanial DOHH since, of those present in the PDB, the structure of YibA was found to be the closest to the leishmanial DOHH. Secondary structural features of *L. donovani* and human DOHH proteins are consistent with the *E. coli* hypothetical protein. In the *Leishmania* protein, there are two significant insertions with respect to the human homolog, corresponding to the alignment positions 159–162 (four amino acid residues) and 174–183 (ten amino acid residues). The *Leishmania* (Figure 2A) and human DOHH protein sequences have been modeled on the crystal structure of the YibA (PDB id, 1OYZ) protein from *E. coli*. The *E. coli* protein is constituted of eight HEAT-repeats forming a horse shoe shaped structure. The *Leishmania* DOHH model superimposes on the template structure with a root mean square deviation of 1.6 Å. The generated model of the pathogen protein shows that the conserved His-Glu motifs are present in the inner circumference of a toroid structure formed by the repeats. Analysis of the generated model also suggests that the amino acid residues that are present in the inner circumference are better conserved than those present in the outer circumference. Moreover, residues that are present near the His-Glu motifs are highly conserved (Figure 2C). This observation supports the proposition that the cavity formed by the His-Glu motifs-containing inner concave surface accommodates the substrate and iron ligands. Conservation scores for all the amino acids of the pathogen protein calculated by ConSurf are given as Information S1. The conservation score indicates the evolutionary rate of each amino acid residue site. High conservation implies high constraints at a particular amino acid residue site due to involvement in enzymatic activity, ligand binding and protein folding or protein-protein interaction. 

Human DOHH was also modeled using E. coli YibA as a template and the generated model superimposed on it with a root mean square deviation of 1.9 Å. The human DOHH modeled structure was compared with the leishmanial DOHH structure (Figure 2D). In the human protein model, the N-terminal His-Glu motifs are present in the structurally equivalent positions, while the C-terminal His-Glu motifs are a little further away from the inner surface, which is suggested to accommodate the substrate [14], [16], [17], as compared to the pathogen protein where the His-Glu motifs are present in the inner circumference of the toroid structure formed by the repeats (Figure 2D). In addition to these topological differences in the C-terminal domain of the human and the parasite proteins, a ten amino acid long insertion is present in the variable loop connecting the N- and C-terminal domains of the generated model of the LdDOHH (Figure 2D) which might affect the specificity of the enzyme for its substrate.

### Overexpression and Purification of Leishmanial Recombinant Deoxyhypusine Hydroxylase in *E. coli*

**Figure 3:**
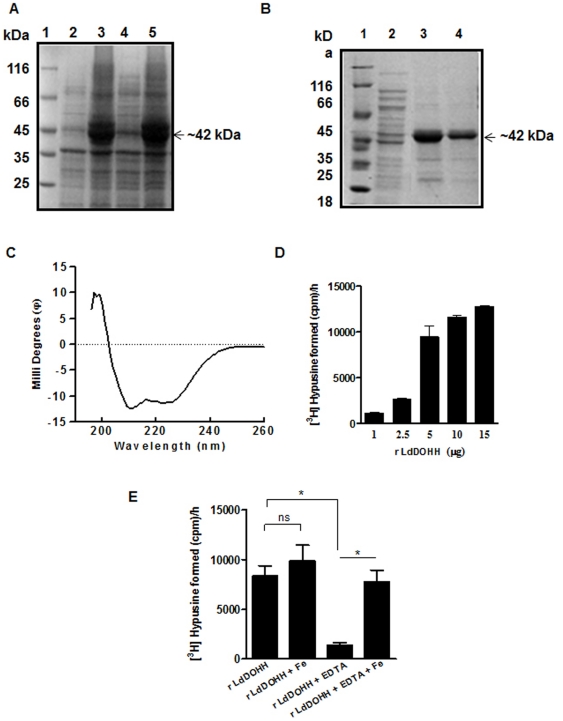
Overexpression, Purification and Characterization of *L. donovani* DOHH protein. (A) Overexpression of DOHH protein after induction at 3 hr with 0.5 mM IPTG. Lane 1, molecular weight marker (MBI Fermentas); Lanes 2 & 4, bacterial cell extract before induction; Lanes 3 & 5, bacterial cell extract after induction (B) Purification of DOHH protein on Ni^2+^-NTA acid affinity resin. Lane 1, molecular weight marker; Lane 2, Flowthrough; Lane 3-4, eluted fractions showing purified DOHH protein with buffer containing 150 mM imidazole. (C) Circular dichroism spectra of the recombinant DOHH showing that it is largely α-helical. The spectra were measured from 260 to 200 nm, at a bandwidth of 1 nm, using 100 µl of solution in a 0.1 mm path length cuvette and the analysis were performed as described in the Materials and Methods. (D) Comparison of the radioactivity in aminopropionaldehyde obtained as a result of [^3^H]hypusine formation after the DOHH assay with 1.0, 2.5, 5.0, 10.0 and 15.0 µg of the recombinant *L. donovani* DOHH protein. The reaction was performed as described in Materials and Methods. Results are mean ± SD of triplicate samples. (E) Comparison of radioactivity in aminopropionaldehyde obtained as a result of [^3^H]hypusine formation after the DOHH assay. The DOHH assay reactions were carried out as described in Materials and Methods. Recombinant DOHH enzyme, purified with and without 4 mM EDTA, was used in the absence and presence of 2 µM ferrous ammonium sulfate. The results are presented are the mean ± SD of triplicate samples. *, *p*<0.05, and ns indicates not significant (p>0.05).

The *DOHH*-pET30a construct was transformed and over expressed in *E. coli* BL21 (DE3) cells. A protein with an estimated molecular weight of ∼42 kDa was induced; the size correlated well with the amino acid composition of DOHH (∼36 kDa) with a 6X-His tag (∼6 kDa) at the C-terminus (Figure 3A). Purification of the DOHH protein by Ni^2+^-NTA-agarose affinity chromatography yielded ∼2 mg of purified protein per liter of the bacterial culture (Figure 3B).

The secondary structure of the recombinant DOHH protein was analyzed by Circular dichroism (CD) spectroscopy. CD spectral analysis of the purified recombinant DOHH revealed that it is highly α-helical, containing 77.7% α-helix (Figure 3C). The α-helical content is very close to that reported for hDOHH (77%) [16] showing that DOHH is a highly conserved protein. This data also correlated with computational modeling of DOHH protein, which suggested that the protein contains eight HEAT repeat motifs and is largely α-helical.

### Deoxyhypusine Hydroxylase Activity

The activity assay for DOHH was performed using human eIF5A ([^3^H]Dhp), obtained by carrying out the deoxyhypusine synthase reaction using human DHS *in vitro,* as the substrate. Figure 3D shows the [^3^H]hypusine formation with increasing concentration of recombinant DOHH. Recombinant DOHH showed a specific activity of ∼117 pmol h^-1^ mg^-1^ of DOHH. This activity was found to be less than 10% of the values reported for the human and the yeast enzymes [14], [16].

The sequence analysis of DOHH revealed that it contains conserved His-Glu motifs which are known to bind to divalent ions. We analyzed the presence of any specific metal ion in the recombinant DOHH, which might be required for its catalytic activity, by inductively coupled plasma-high resolution mass spectrometry (ICP-MS). Iron was found to be the major metal. Its content was found to be ∼1.2 mol of iron/ mol of DOHH. Iron content was also measured in the recombinant LdDOHH that had EDTA (4 mM) in the sonication buffer. The iron content was found to be 0.5 mol of iron /mol of LdDOHH whereas the hDOHH under similar condition had <0.07 mol of iron /mol of hDOHH. The content of other metals like zinc and magnesium was also analyzed. A very low level of zinc was found (0.034 mol/mol), whereas magnesium could not be detected in the recombinant DOHH protein. To further confirm that iron was indeed important for the activity of recombinant DOHH, the activity assays of leishmanial DOHH were performed in the presence and absence of iron as described in the Materials and Methods. No significant increase in recombinant leishmanial DOHH activity was observed upon addition of ferrous ion as compared to the activity of DOHH without the ferrous ions (Figure. 3E). However, the enzyme when purified with EDTA in its sonication buffer showed a marked reduction in the DOHH activity. The addition of ferrous ion to this preparation restored its activity significantly, indicating that the depletion of iron was the cause for the reduced activity of the protein (Figure 3E). It thus further confirmed that the leishmanial DOHH is an iron-binding enzyme and requires iron for its catalytic activity.

### Functional Analysis of *L. donovani* Mutant Recombinant DOHH enzyme

**Figure 4:**
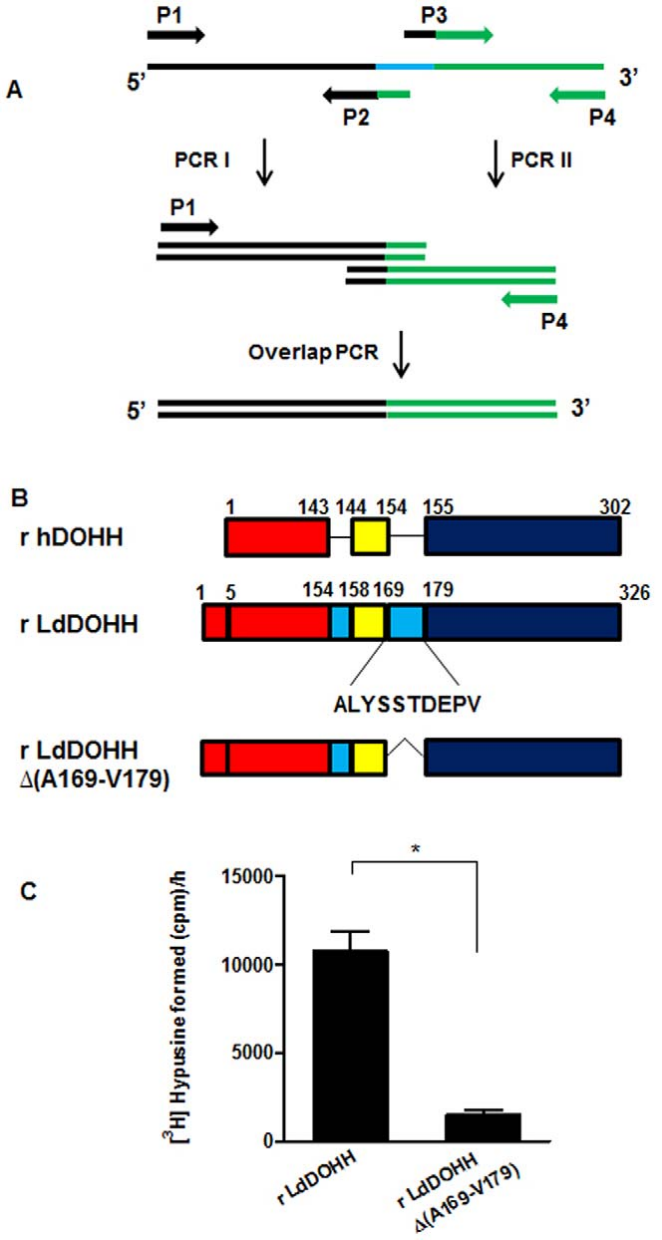
Effect of deletion mutation on the activity of *L. donovani* DOHH. (**A**) Scheme representing overlap extension PCR used for deletion of the ten-amino-acid-long insertion from LdDOHH. The regions shown in black and green are the regions upstream and downstream of the region to be deleted (shown in cyan blue). (**B**) Alignment showing human DOHH, LdDOHH and LdDOHH ▵(A169-V179). The regions that are similar between hDOHH and LdDOHH and align with each other are shown in red, yellow and blue color. Cyan blue color shows the insertions present in LdDOHH. (**C**) Comparison of the radioactivity in aminopropionaldehyde obtained as a result of [^3^H]hypusine formation after the DOHH assay with the recombinant LdDOHH enzyme or the mutant LdDOHH ▵(A169-V179) enzyme. The results presented are the mean ± SD of triplicate samples. *, *p*<0.05.

Sequence analysis revealed the presence of a ten-amino-acid-long insertion at the position 169–179, in the loop connecting the N-terminal and C-terminal His-Glu motifs. A representative figure of hDOHH and the insertions in LdDOHH is shown in Figure 4B. The role of this insertion in *L. donovani* DOHH was assessed by deletion mutagenesis. A construct LdDOHH ▵(A169-V179) was created by overlapping extension PCR and the protein was expressed and purified as described in the Materials and Methods. Interestingly, the activity of the mutant enzyme was reduced to ∼14% of the wild type recombinant enzyme, indicating that the insertion is crucial for the activity of recombinant LdDOHH (Figure 4C).

### Effect of Inhibitors on DOHH Activity of *L. donovani*

In our earlier work we have shown that the inhibitors known for the hypusine pathway in humans are not effective against *Leishmania* which makes its hypusine pathway unique [21]. Despite conservation of some of the active site amino acid residues between the human and leishmanial DHS, a potent inhibitor of human DHS, *N* ^1^-guanyl-1, 7-diaminoheptane, had little inhibitory effect on either *L. donovani* proliferation or recombinant DHS34. To assess the significance of deoxyhypusine hydroxylation in *L. donovani*, the effect of known inhibitors of DOHH was examined on the enzymatic activity of DOHH *in vitro*. There are no specific inhibitors available for DOHH to date. However, it is known that, like other hydroxylases, DOHH is also inhibited by certain metal chelators [22], [23]. The inhibitors used were the metal chelators, mimosine, CPX and kojic acid. Mimosine, a plant alkaloid, has been shown to inhibit the enzyme DOHH causing cell cycle arrest at the G1-S stage in Chinese hamster ovary cells [22]. CPX is a topical anti-fungal and is known to block DOHH activity [23]. Kojic acid has a metal chelating domain identical to that of mimosine [22].

**Figure 5:**
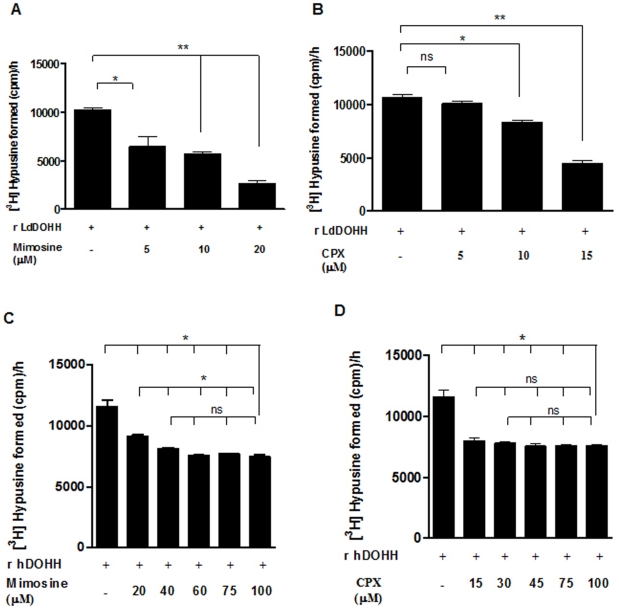
Effect of mimosine and CPX on deoxyhypusine hydroxylase activity. Recombinant LdDOHH (5 µg) was incubated with either (A) mimosine (5, 10 or 20 µM) or (B) CPX (5, 10 and 15 µM) for 10 min at 37°C. Recombinant hDOHH was incubated with varying concentrations of (C) mimosine (20, 40, 60, 75 and 100 µM) and (D) CPX (15, 30, 45, 75 and 100 µM) for 10 min at 37°C. [^3^H]Hypusine formation was then measured as detailed in the Methods section. Results are mean ± SD of triplicate samples. *, *p*<0.05; **, *p*<0.002 and ns indicates not significant (p>0.05).

We investigated the effect of these inhibitors on the activity of the recombinant deoxyhypusine hydroxylase from *L. donovani*. The antifungal compound, kojic acid, was found to be ineffective in the inhibition of DOHH activity in *L. donovani* (Data not shown). A range of concentrations of both mimosine (5 µM, 10 µM or 20 µM) and CPX (5 µM, 10 µM or 15 µM) was used against *L. donovani* recombinant DOHH. Mimosine (20 µM) and CPX (15 µM) resulted in 70% and 60% inhibition of *L. donovani* DOHH activity, respectively when compared to the control untreated group (Figure 5A and B). Mimosine (15 µM) and CPX (15 µM) resulted in only ∼22% and ∼32% inhibition of the recombinant human DOHH (Figure. 5C and D). Interestingly, both the inhibitors were more effective against the leishmanial enzyme as compared to the human enzyme (Figure 5C and D). Mimosine, (15 µM) inhibited the activity of LdDOHH and hDOHH by 60% and 22% respectively (Figure 5C). On the other hand, CPX (15 µM) was found to be more effective at the same concentration and was able to inhibit LdDOHH activity by 60% and decreased hDOHH activity by only 32% compared to the untreated recombinant DOHH activity (Figure 5D). The inhibitor data obtained was fitted onto the Hill equation curve to determine the concentration of the inhibitor at which 50% of enzymatic activity was inhibited. The concentration of mimosine and CPX that inhibited 50% of the LdDOHH activity were 13 µM and 13.6 µM respectively. On the other hand, when a range of concentrations of mimosine and CPX were used against recombinant hDOHH, a maximum of ∼35% of hDOHH activity was inhibited with concentrations as high as 100 µM (Figure 5C and D).

We then examined the effect of the inhibitors, mimosine, CPX and kojic acid, on the cell growth of *L. donovani* promastigotes and intracellular amastigotes. The antifungal compound, kojic acid, was found to be ineffective in the inhibition of the growth of both AG83 promastigotes and amastigotes, even at concentration as high as 1 mM (data not shown). The concentration of mimosine as high as 500 µM, inhibited the cell proliferation of promastigotes by only 30% (Table 1). On the other hand, CPX was found to be highly effective; the concentration of CPX that inhibited the growth of WT promastigotes by 50% was ∼2.1 µM (Table 1). The effect of these inhibitors was also checked on the intracellular amastigote form (which is the more relevant stage of the parasite). The concentration of CPX and mimosine that inhibited the growth of WT amastigotes by 50% was ∼0.2±0.03 µM and 1.09±0.31 µM respectively. Both CPX and mimosine at these concentrations did not affect the viability of the macrophage cell line THP1, the IC_50_ being >10 and 40 µM respectively.

**Table 1:** Effect of mimosine and CPX on promastigotes, amastigotes of *Leishmania donovani* and on human macrophage-like cell line THP-1.

	IC_50_ (µM)		
Inhibitor	Promastigotes	Intracellular Amastigotes	Uninfected Macrophages
Mimosine	>500	1.09±0.31	>40
CPX	2.14±0.21	0.2±0.03	>10

IC_50_s were determined 72 h after drug addition as reported in the Materials section.

Results are Mean ± Standard deviation of triplicate values.

## Discussion

[TIGHEST]In the present study, we report for the first time the identification and characterization of deoxyhypusine hydroxylase from *L. donovani*. Sequence alignment shows that *L. donovani* DOHH shares∼16% and ∼40.6% sequence identity with the *E. coli* hypothetical protein YibA and human DOHH respectively (Figure 1). *Leishmania* and human DOHH protein sequences have been modeled on the crystal structure of YibA protein from *E. coli*. The parasite and human protein models show that they are both HEAT-repeat proteins containing eight metal coordination sites consisting of four strictly conserved His-Glu sequences. While 3-D structural superposition of the *L. donovani* and human DOHH proteins suggests that the His-Glu motifs of the two proteins are located in structurally equivalent positions in the N-terminal domain, these sequentially conserved motifs are not quite equivalent in the C-terminal domain (Figure 2D) [17]. Furthermore, sequence insertions that have been found at two places in *L. donovani* are probably in the variable loop connecting the N- and C-terminal domains of the protein (Figure 2D). These 3-D structural deviations in the C-terminal domain along with the ten-amino-acid-residue-long insertion in the inner concave surface of generated model might account for the differences in the inhibitor binding properties of the parasite enzyme from that of the human homolog. Deletion of this ten-amino-acid-long insertion reduced the DOHH activity of the enzyme to 14% of the activity of the wild type, indicating that it is critical for its activity (Figure 4C).

A single gene for DOHH is present in all eukaryotic organisms and is quite conserved. It is absent from archaea and bacteria, though *DHS*-like genes are found in archaea and certain bacteria [11]. Deoxyhypusine hydroxylase belongs to a family of proteins that contain tandem repeats of HEAT motifs. Human DOHH is composed of two symmetrical domains, each containing four HEAT-repeats. It depends on Fe^2+^ ions for its activity and has four strictly conserved His-Glu pairs that act as metal co-ordination sites [14]. The residues His-56, His-89, Glu-90, His-207, His-240, and Glu-241 of human DOHH have been identified as iron coordination sites. Sequence analysis of *L. donovani* DOHH showed low sequence identity with the human homolog. It also contains eight tandem HEAT repeats, four in each N- or C- terminal domain. *Plasmodium* DOHH is an exception as it has been reported to have 5 HEAT-repeats instead of eight [24]. Sequence analysis of the *L. donovani* DOHH indicates the presence of four His-Glu motifs corresponding to the residues present in the human homolog. These observations suggest that *L. donovani* DOHH is also a metalloenzyme. Therefore, we studied the effect of metal chelators on both the activity of *L. donovani* recombinant DOHH and cell growth. Mimosine and CPX strongly inhibited the activity of recombinant DOHH *in vitro*. Interestingly, we found that both the compounds inhibited the leishmanial DOHH more effectively as compared to the human counterpart. The differences in sensitivity could be due to differences in the interactions of the compounds with each of the enzymes and may be exploited for anti-parasitic effects. The inhibition of enzymatic activity of DOHH *in vitro* by CPX correlated well with the inhibition of cell proliferation of AG83 promastigotes as well as intracellular amastigotes (Table 1). Mimosine, which inhibited recombinant DOHH activity *in vitro*, also inhibited the cell growth of intracellular amastigotes effectively (IC_50_ = 1.09±0.31 µM). In contrast, it was a poor inhibitor of the growth of promastigotes, even at a much higher concentration (IC_50_ >500 µM). The reason for the difference may be due to differences in the efficiency of their uptake into the cells. Since mimosine and CPX are metal chelators and not specific inhibitors for DOHH enzyme, it is possible that they may have off-target effects in the *Leishmania* parasites and might inhibit other enzymes as well, leading to inhibition of cell growth of promastigotes and intracellular amastigotes. However, both CPX and mimosine, at concentrations 10 and 40 µM, respectively, did not show any effect on uninfected human macrophage-like THP-1 cells *in vitro.* Kojic acid, whose metal chelating domain is identical to mimosine, failed to show any effect on DOHH activity *in vitro* or growth (data not shown). This observation correlates with earlier studies establishing that the compounds must have a planar ring and an amino side chain to effectively inhibit DOHH [22].

DOHH is the second enzyme in the hypusine pathway and catalyzes the maturation of eIF5A, which has been found to be an essential protein in cell proliferation. Inhibitors of DOHH have been shown to have anti-proliferative effects in mammalian cells, including cancer cells, and lead to cell cycle arrest, indicating the importance of hypusine modification in eukaryotic cells [22]. Metal chelating inhibitors of DOHH, such as deferiprone and CPX, have been shown to have anti-retroviral effects by inhibiting DOHH activity in cells thereby depleting cellular eIF5A levels and ultimately affecting mRNA translation [19]. Therefore, eIF5A and DOHH have been proposed as targets for anti-tumor and anti-retroviral chemotherapy [19], [20]. Molecular cloning of DOHH from *L. donovani* indicates that the entire hypusine biosynthetic pathway exists in this organism. We have earlier demonstrated that the first enzyme of the pathway, DHS, plays a vital role in survival and proliferation of *L. donovani* [21] and it is probale that *DOHH* is also vital in *L. donovani.* However, DOHH unlike DHS is not essential in *S. cerevisiae* [14]. Whether the leishmanial DOHH is essential or not can not be assumed at this stage and needs further verification.

The ability to regulate cell growth by inhibition of DOHH may provide us with a chemotherapeutic target. Our results demonstrate that both CPX and mimosine exhibited selective activity against the pathogen and not against the host. Further studies are required to investigate the structure of the enzyme and its mechanism of action in order to develop selective inhibitors against the parasite.

## Materials and Methods

### Chemicals

Radiolabeled spermidine trihydrochloride [1,8-^3^H]spermidine (32.35 Ci/mmol) was purchased from PerkinElmer Life Sciences. MTT, [3-(4,5-dimethylthiazol-2-yl)-2, 5-diphenyltetrazolium bromide] was purchased from Sigma (St. Louis, MO). CENTA^TM^, β-lactamase substrate was purchased from Calbiochem (La Jolla, CA). The compounds mimosine, CPX and kojic acid were purchased from Sigma Aldrich (St. Louis, MO). All restriction enzymes and DNA modifying enzymes were obtained from MBI Fermentas (Germany). The other materials used in this study were of analytical grade and were commercially available.

### Parasite and Culture Conditions

*L. donovani* AG83 (MHOM/IN/1983/AG83) promastigotes were cultured at 22°C in modified M199 medium (Sigma) supplemented with 100 U/ml penicillin (Sigma), 100 μg/ml streptomycin (Sigma) and 10% heat inactivated fetal bovine serum (FBS, Hyclone).

### Effect of Inhibitors on the Growth of Promastigotes of *L. donovani*

The effect of inhibitors of DOHH on the growth of *L. donovani* AG83 promastigotes was determined by the MTT assay. 25 µl of 1×10^6^ parasites/ml of mid log phase promastigotes were cultured in 96 well tissue culture plate and incubated with 25 µl of varying concentrations of CPX, mimosine and kojic acid at 22°C. After 72 h, 20 µl of 5 mg/ml MTT [3-(4, 5-dimethylthiazol-2-yl)-2, 5-diphenyltetrazolium bromide] dissolved in phosphate buffered saline (pH 7.2) was added. Plates were incubated at 37°C until purple-colored crystals were formed. The reaction was stopped by the addition of 50 µl of 50% isopropanol and 10% sodium dodecyl sulfate with gentle shaking at 37°C for 1 h. Absorbance was measured spectrophotometrically at 570 nm.

### Intracellular Amastigote-Macrophage Cultures and Drug Effect

THP-1 human macrophage-like cell line was obtained from ATCC and maintained in RPMI- 1640 medium (Sigma) supplemented with 10% FBS at 37°C in 5% CO_2_ atmosphere. Before infection, 200 µl of 5×10^5^ cells/ml cells were plated in 96-well plates and differentiated with phorbol myristic acetate (PMA) (20 ng/ml). They were allowed to adhere for 48 h and were infected with stationary phase AG83 promastigotes transfected with the β-lactamase gene at a ratio of 20 parasites per monocyte, as reported earlier [25]. After 6 h of infection, the non-internalized parasites were washed off with RPMI medium and different concentrations of drugs were added. Intracellular amastigotes grown in THP-1 cell lines were quantified after 72 h of drug addition for β-lactamase activity by first removing the medium by gentle pipetting. Subsequently, 50 µl of 50 µM CENTA^TM^ in 1X PBS and 0.1% Nonidet P-40 were added. The plates were incubated at 37°C for 4 h and the absorbance was read at 405 nm.

### Cloning of Putative Deoxyhypusine Hydroxylase Gene (DOHH) from *L. donovani*

A 981- bp DNA fragment encompassing the whole open reading frame (ORF) of *L. donovani DOHH* gene was amplified from the genomic DNA using a sense primer with a flanking *Eco*RI site, 5′- CGGAATTC ATGTCTGCTTTGAACAGCCGCACCGTCGA-3′ and an antisense primer with a flanking *Hin*dIII site, 5′-CCCAAGCTTCCTGTTGAACACCCCTCTCACGCCTCCTG-3′. The amplified product was obtained and subcloned into the pTZ57R/T (MBI Fermentas) vector and subjected to automated sequencing.

### Construction of *L. donovani* Deoxyhypusine Hydroxylase Mutant Gene

Sequence analysis revealed the presence of a ten-amino-acid long insertion at the position 169-179 in the loop connecting the N-terminal and C-terminal His-Glu motifs. To study the importance of this insertion in the *L. donovani* DOHH protein, deletion mutagenesis was performed by the overlap extension PCR method as described by [26]. The basic scheme of the PCR is described in Figure 4A. The PCR primers used were: P1: 5′- CGGAATTC ATGTCTGCTTTGAACAGCCGCACCGTCGA-3′; P2: 5′- GGTGTGCGG GCTAAACGCCGGCGACGGATCCACAGAC-3′; P3: 5′-GCGTTTAGC CCGCACACCGTGGAGGAACTGGAG-3′; P4; CCCAAGCTTCCTGTTGAACACCCCTCTCACGCCTCCTG-3′. The final PCR product was obtained and cloned into pET30a vector (Novagen) and subjected to automated sequencing.

### Sequence and Structure Analysis

In order to facilitate the comparative analysis of DOHH protein sequences from diverse eukaryotic organisms, the DOHH protein sequence from *L. donovani* was searched using the PSI-BLAST approach [27] in the Uniref90 sequence database, which is a comprehensive collection of protein sequences from diverse organisms (www.ebi.ac.uk/uniref/). The CD-HIT program [28] was used to generate a non-redundant dataset of homologous DOHH sequences with the sequence identity cut off of 90%. The multiple sequence alignment program CLUSTALW [29] was used to align DOHH sequences from different sources. A phylogenetic tree was generated using a neighbor-joining (NJ) method [30]. The MEGA program (version 4.0) was used to draw the tree [31]. The tree was annotated with bootstrap values (1000 iterations). The Program JOY [32] was used for displaying the three-dimensional structural features of the human DOHH and *Leishmania* DOHH protein sequences with respect to the crystal structure of the *E. coli* YibA protein.

Sequence analysis-based recognition of homologs of DOHH was confirmed using the fold recognition approach PHYRE (Protein Homology/analogY Recognition Engine) version 0.2 [33]. PHYRE fitted the pathogen DOHH sequence on the structure of the hypothetical *Escherichia coli* protein, YibA (PDB accession code: 1OYZ) with very high reliability (estimated precision 100%) and reliable E-value (10^-18^). *Leishmania* and human protein sequences were modeled on the tertiary structure of the *E. coli* protein template using MODELLER version 9.0 (Sali Lab, UCSF, San Francisco, CA, USA, http://www.salilab.org/modeller) [34]. The generated models were energy minimized using Kollman united atom forced field in SYBYL [Tripos Inc., St. Louis, MO, USA] to relieve short contacts, if any.

The generated models were superimposed on a template structure by using DaliLite program [35]. The overall fit of the sequence to the modeled structure was checked using PROSA [36], [37]. The stereochemistry of the energy-minimized models was ensured using PROCHECK [38], [39]. The *Leishmania* DOHH protein model thus generated was analyzed using ConSurf (Conservation Surface Mapping Method) [40], which maps the conserved regions on the surface of a protein structure based on phylogeny among related sequences. Structural visualization was performed using the PYMOL program {DeLano W L 83 /id}[41]. 3-D structural models of the human protein and *L. donovani* are compared using the DaliLite program [31]. Structural models superposed with a Z-score of 29.2 and an RMSD of 4.8 Å. The structure-based sequence identity of the human and parasite protein is 18%.

### Construction of *L. donovani* DOHH Expression Vector and Purification of the recombinant and the mutant DOHH Protein

The coding region of *DOHH* gene was subcloned into *Eco*RI-*Hin*dIII site of pET30a vector (Novagen). The fidelity of the PCR-amplified *L. donovani DOHH* was confirmed by automated DNA sequencing. The constructs containing either wild type DOHH or mutant DOHH were transformed into the BL21 (DE3) strain of *E. coli*. Protein expression for both the constructs was induced by 0.5 mM isopropyl-1-β-d-galactopyranoside (IPTG) at 37°C for 3 h. Bacteria were then harvested by centrifugation at 5000×*g* for 10 min and the cell pellet was resuspended in binding buffer (50 mM Tris.Cl pH 7.5, 10 mM imidazole, 300 mM sodium chloride, 1 mM phenylmethylsulfonyl fluoride (PMSF) and 30 μl of protease inhibitor cocktail (Roche Applied Sciences, Germany). The resulting cell suspension was sonicated six times for 15 s with 1 min intervals. The lysate was centrifuged at 10,000×*g* for 30 min at 4°C. The resulting supernatant, which contained the protein, was loaded onto pre-equilibrated Ni^2+^-Nitrilotriacetic acid (NTA) – agarose resin (Qiagen). The mixture was kept on a rocking platform for 2 h at 4°C. It was centrifuged at 400×*g* for 1 min at 4°C. The supernatant was removed and the resin was washed three times with wash buffer (50 mM Tris.Cl, pH 7.5, 20 mM imidazole, 300 mM NaCl, 1 mM PMSF and protease inhibitor cocktail). The proteins were eluted with increasing concentrations of imidazole. Imidazole was removed by dialysis and the purified proteins were found to be >95% pure as judged by SDS-PAGE gel.

For metal analysis, a large scale preparation of the enzyme was carried out. Two preparations of the recombinant protein were made: one as described above and the second preparation included 4 mM EDTA in the sonication buffer. The buffers used for purification were made in metal-free HPLC water. The purification procedure followed was the same as described above.

### Purification of Human Recombinant Deoxyhypusine Hydroxylase

The plasmid construct encoding the human enzyme hDOHH-pGEX-4T-3 was transformed into *E. coli* BL21 cells and the protein was over expressed and purified with a GST tag as described earlier [16]. The purified protein was then treated with the RECOMT Thrombin CleanCleave™ Kit (Sigma) and the GST tag was removed according to the manufacturer’s protocol.

### Analysis of Secondary Structure of DOHH by Circular Dichroism

Circular dichroic spectra were measured in a Chirascan™ circular dichroism spectrometer. Far ultraviolet spectra were measured from 260 to 200 nm, bandwidth of 1 nm, using 100 µl of solution in a 0.1 mm path length cuvette. The protein concentration was measured spectrophotometrically and used at 0.6 mg/ml. Data were obtained in milli degrees and converted into Delta Epsilon (Δε) for estimation of secondary structure using CDNN software using a mean residue weight of 113.

### Preparation of Radiolabeled eIF5A (Dhp) Substrate for DOHH Assay

Polyhistidine-tagged human eIF5A and human deoxyhypusine synthase used for the preparation of radiolabeled eIF5A (Dhp) substrates were purified as described [42]. The human eIF5A([^3^H]Dhp) was prepared by an *in vitro* deoxyhypusine synthase reaction using [^3^H]spermidine as described previously [43]. Since human eIF5A(Dhp) acts as an effective substrate for *Leishmanial* DOHH, it was used for the assay of the *Leishmanial* enzyme.

### Deoxyhypusine Hydroxylase Assay

The activity of the recombinant purified deoxyhypusine hydroxylase was measured as the formation of radioactive aminopropionaldehyde after periodate oxidation of the hypusine-containing product, eIF5A([^3^H]Hpu), formed following the DOHH reaction [44]. A typical DOHH reaction mixture contained 20 mM Tris.Cl pH 7.5, 6 mM DTT, 1 mg/ml BSA, radiolabeled protein substrate, human eIF5A ([^3^H] Dhp) (2-4×10^4^ cpm) and 1-15 µg of purified enzyme in 40 µl of total volume. After incubation in a 37°C water bath for 2 h, the reaction mixture was divided into two halves. To the first half of the reaction mixture, 250 µg of BSA was added followed by precipitation with 100 µl of 10% trichloroacetic acid. To the other half of the reaction mixture, 40 µl of 0.2 M sodium phosphate/ citrate buffer, pH 6.4, and 15 µl of 0.3 M sodium meta-periodate were added and kept at RT for 2 h. The reaction was stopped by adding 250 µg of BSA followed by precipitation with 200 µl of 10% TCA. The TCA precipitates from both reactions were centrifuged at 15,000×*g* at 4°C and the counts were recorded with a liquid scintillation counter. The radioactivity in the TCA supernatant of an unoxidized sample was subtracted from that of the oxidized sample to calculate the net hypusine formed [44]. The assay was performed in the presence or absence of an inhibitor. The reaction mixture was pre-incubated with varying concentrations of mimosine or CPX at 37°C for 10 min and then the substrate, eIF5A([^3^H]Dhp), was added and the reaction was carried out in a 37°C water bath for 2 h. The rest of the assay was performed as described above.

### Analysis of the Metal Content of DOHH

The buffers for the recombinant LdDOHH enzyme were prepared using metal-free HPLC water. The protein samples were analyzed for metal content (Iron, magnesium and zinc) by inductively coupled plasma-high resolution mass spectrometry (ICP-MS) (ARBRO Pharmaceuticals Ltd, New Delhi, India). To further confirm if iron is required for the DOHH activity, activity assays were performed with two different DOHH preparations. The enzyme preparation was done in the presence and absence of 4 mM EDTA in the sonication buffer. The activity assays were performed in the presence and absence of ferrous ammonium sulphate (2 µM). The DOHH enzyme assay was set up as described above.

### Statistical Analysis

T-test was performed using Graph-Pad Prism version 5.0 for Windows to determine the P- values. Values of *p*<0.05 were considered statistically significant. Data are presented as mean ± SD.

## Supporting Information

Figure S1 **Multiple sequence alignment of deoxyhpusine hydroxylase protein sequences from** ***Leishmania donovani*** **along with its eukaryotic homologs.** Conserved His-Glu motifs are highlighted in yellow. Apart from the His-Glu motifs, other absolutely conserved residues are highlighted in gray. Accession numbers and the corresponding organism as the source of the DOHH used in generating the multiple sequence alignment are as follows: B0S4Z5_DANRE: *Danio_rerio*, B0W942_CULQU: *Culex_quinquefasciatus*, B2WFV6_PYRTR: *Pyrenophora_tritici-repentis*, B6K221_SCHJY: *Schizosaccharomyces japonicus*, B9WC15_CANDC: *Candida_dubliniensis*, C0SAR9_PARBP: *Paracoccidioides brasiliensis*, C1BJA2_OSMMO: *Osmerus mordax*, C1BQB5_9MAXI: *Caligus rogercresseyi*, C1BVM4_9MAXI: *Lepeophtheirus salmonis*, C4R113_PICPG: Pichia pastoris, C5FM15_NANOT: *Nannizzia otae*, C5K274_AJEDS: *Ajellomyces dermatitidis*, C8VBH9_EMENI: *Aspergillus nidulans*, C9QNK6_PLAFO: *Plasmodium falciparum*, D0NC43_PHYIN: *Phytophthora infestans*, DOHH1_ORYSJ: *Oryza sativa subsp. japonica*, DOHH_ARATH: *Arabidopsis thaliana*, DOHH_ASHGO: *Ashbya gossypii*, DOHH_ASPCL: *Aspergillus clavatus*, DOHH_ASPFU: *Aspergillus fumigatus*, DOHH_ASPNC: *Aspergillus niger*, DOHH_ASPOR: *Aspergillus oryzae*, DOHH_BOVIN: *Bos taurus*, DOHH_CAEEL: *Caenorhabditis elegans*, DOHH_CANGA: *Candida glabrata*, DOHH_CHAGB: *Chaetomium globosum*, DOHH_CHICK: *Gallus gallus*, DOHH_COCIM: *Coccidioides immitis*, DOHH_CRYNE: *Cryptococcus neoformans*, DOHH_DEBHA: *Debaryomyces hansenii*, DOHH_DICDI: *Dictyostelium discoideum*, DOHH_DROME: *Drosophila melanogaster*, DOHH_ENCCU: *Encephalitozoon cuniculi*, DOHH_GIBZE: *Gibberella zeae*, DOHH_HUMAN: *Homo sapiens*, DOHH_KLULA: *Kluyveromyces lactis*, DOHH_LENED: *Lentinula edodes*, DOHH_MOUSE: *Mus musculus*, DOHH_NEUCR: *Neurospora crassa*, DOHH_PHANO: *Phaeosphaeria nodorum*, DOHH_SCHPO: *Schizosaccharomyces pombe*, DOHH_USTMA: *Ustilago maydis*, DOHH_XENLA: *Xenopus laevis*, DOHH_YARLI: *Yarrowia lipolytica*, DOHH_YEAST: *Saccharomyces cerevisiae*, Q4Q901_LEIMA: *Leishmania major*, A4I2C0_LEIIN: *Leishmania infantum* and Q38FR2_9TRYP: *Trypanosoma brucei*.
(PDF)

Figure S2 **Phylogenetic analysis of DOHH protein sequences from different eukaryotic sources.** The phylogram presented is a consensus of 1000 bootstrap replicates constructed using the MEGA program (Ver. 4.0). The numbers at the node present the percentage of trees with the same node among all the bootstraps. *L. donovani* DOHH protein sequence clusters with other eukaryotic pathogens such as *Plasmodium falciparum* and *Trypanosoma brucei*.
(PDF)

Information S1 Amino Acid Conservation Scores.
(PDF)
