# Supplementary material for: A Unique Modification of the Eukaryotic Initiation Factor 5A Shows the Presence of the Complete Hypusine Pathway in Leishmania donovani
Source: PLoS One. 2012 Mar 16;7(3):e33138. doi: 10.1371/journal.pone.0033138 (PMC3306375; doi:10.1371/journal.pone.0033138)
Supplement: Figure S1 — Multiple sequence alignment of deoxyhpusine hydroxylase protein sequences from Leishmania donovani along with its eukaryotic homologs. Conserved His-Glu motifs are highlighted in yellow. Apart from the His-Glu motifs, other absolutely conserved residues are highlighted in gray. Accession numbers and the corresponding organism as the source of the DOHH used in generating the multiple sequence alignment are as follows: B0S4Z5_DANRE: Danio_rerio, B0W942_CULQU: Culex_quinquefasciatus, B2WFV6_PYRTR: Pyrenophora_tritici-repentis, B6K221_SCHJY: Schizosaccharomyces japonicus, B9WC15_CANDC: Candida_dubliniensis, C0SAR9_PARBP: Paracoccidioides brasiliensis, C1BJA2_OSMMO: Osmerus mordax, C1BQB5_9MAXI: Caligus rogercresseyi, C1BVM4_9MAXI: Lepeophtheirus salmonis, C4R113_PICPG: Pichia pastoris, C5FM15_NANOT: Nannizzia otae, C5K274_AJEDS: Ajellomyces dermatitidis, C8VBH9_EMENI: Aspergillus nidulans, C9QNK6_PLAFO: Plasmodium falciparum, D0NC43_PHYIN: Phytophthora infestans, DOHH1_ORYSJ: Oryza sativa subsp. japonica, DOHH_ARATH: Arabidopsis thaliana, DOHH_ASHGO: Ashbya gossypii, DOHH_ASPCL: Aspergillus clavatus, DOHH_ASPFU: Aspergillus fumigatus, DOHH_ASPNC: Aspergillus niger, DOHH_ASPOR: Aspergillus oryzae, DOHH_BOVIN: Bos taurus, DOHH_CAEEL: Caenorhabditis elegans, DOHH_CANGA: Candida glabrata, DOHH_CHAGB: Chaetomium globosum, DOHH_CHICK: Gallus gallus, DOHH_COCIM: Coccidioides immitis, DOHH_CRYNE: Cryptococcus neoformans, DOHH_DEBHA: Debaryomyces hansenii, DOHH_DICDI: Dictyostelium discoideum, DOHH_DROME: Drosophila melanogaster, DOHH_ENCCU: Encephalitozoon cuniculi, DOHH_GIBZE: Gibberella zeae, DOHH_HUMAN: Homo sapiens, DOHH_KLULA: Kluyveromyces lactis, DOHH_LENED: Lentinula edodes, DOHH_MOUSE: Mus musculus, DOHH_NEUCR: Neurospora crassa, DOHH_PHANO: Phaeosphaeria nodorum, DOHH_SCHPO: Schizosaccharomyces pombe, DOHH_USTMA: Ustilago maydis, DOHH_XENLA: Xenopus laevis, DOHH_YARLI: Yarrowia lipolytica, DOHH_YEAST: Saccharomyces cerevisiae, Q4Q901_LEIMA: Leishmania major, A4I2C0_LE [file pone.0033138.s001.pdf]

```
B0S4Z5_DANRE      ---------------------------------MAN----DKDIAAVGSILVNTK--QDLTTRFRALFTLRNLGGA---------EAVKWISEAFV-DESALLKHELAYCLGQMQDESAI
C1BJA2_OSMMO      ---------------------------------MAS----DQEVAAVGQILANGK--QDLSTRFRALFTLRNLGGA---------EAICWISKTFE-DESALLKHELAYCLGQMQDERAI
DOHH_BOVIN        ---------------------------------MVT----EQEVEAVGQTLVDPG--QPLQARFRALFTLRGLGGP---------VAISWISRAFD-DDSALLKHELAYCLGQMQDRRAI
DOHH_HUMAN        ---------------------------------MVT----EQEVDAIGQTLVDPK--QPLQARFRALFTLRGLGGP---------GAIAWISQAFD-DDSALLKHELAYCLGQMQDARAI
DOHH_MOUSE        ---------------------------------MVT----EQEIEAIGKTLVDPK--QPLQARFRALFTLRGLGGP---------DAISWISRGFE-DSSALLKHELAYCLGQMRDARAI
DOHH_CHICK        ---------------------------------MVT----EEEVTAIGRTLLDAA--QPLPARFRALFTLRNLGGP---------AAIDCIVRGFA-DSSALLKHELAFCLGQMRDRAAI
DOHH_XENLA        ---------------------------------MAASL--SSEVHSLGQLLIDPG--KPLPLRFRALFTLRNLGGA---------EAIDCIGRGFQ-DESALLKHELAYCLGQMKDRRAL
B0W942_CULQU      -------------------------------MVQIE----ESKIADIGRVLNDKD--RPLKERFRALFTLKNIGGP---------SALASIESCFD-DESALLKHELAYCLGQMQDRAAI
DOHH_DROME        ---------------------------------MVS----QQQIEAIGGVLNNKE--RPLKERFRALFTLKNIGGG---------AAIEAISKAFD-DDSALLKHELAYCLGQMQDAQAL
DOHH_CAEEL        ----------------------------MVASQKFS----DAEIDSFGEALNDTK--KPLKARFRALFILRNIGCD---------RSVDWIGKCLN-DESALLKHELAYCLGQMQNKHAI
B2WFV6_PYRTR      ----------------------------MAVTEQDT------QVSTLRNILSSES--EPLARRFRALFSLKHLAGLQPPN-AQTVPAIEAIAAAFS-SPSALLKHELAYCLGQSGHDAAI
DOHH_PHANO        ----------------------------MAVTEQDT------QVPTLRKIVTSES--EPLARRFRALFSLKHLASLQPPT-EQTVPAIEAIAAAFS-SPSALLKHELAYCLGQSGHDAAI
C0SAR9_PARBP      ------------------------MNQPSNEGADSS------PIPTLGKTLISEK--EPLARRFRALFSLKHIACQQPPT-EENLPAIQAIAAAFT-SSSALLKHELAYCLGQTKNLACV
C5K274_AJEDS      -----------------MPSTAIDTPKPSSMGASSS------PIPPLRKTLTSEN--EPLARRFRALFSLKHVACLQPPT-EQSLHAIQAIAAAFS-SPSALLKHELAYCLGQTKNLAAV
C5FM15_NANOT      -------------------MANDGTNGNTQIDNVSQ------TVKALRDTLTSET--TPLARRFRALFSLKHLACLQPPT-DDTIPAIEAIAAAFP-SPSALLKHELAYCLGQTKNPHAI
DOHH_ASPCL        -------------------MSPSATDNSDG---PDA------TVLTLRKVLTSES--EPLARRFRALFSLKYLACQQPAT-EKTLPAIQAIAAAFT-SPSALLKHELAYCLGQTRNPESV
DOHH_ASPFU        -------------------MSPSATDTTEV---MDP------TILTLRKVLTSES--EPLGRRFRALFSLKHLACLQPPT-EKTLPAIQAIAAAFT-SPSALLKHELAYCLGQTRNPASL
DOHH_ASPNC        -------------------MSAVNENNLEG---VSE------TVLTLRKVVVNES--EPLARRFRALFSLKYLACLQPPS-EDTLPAIEAIAAAFS-SKSALLKHELAYCLGQTRNPDAV
DOHH_ASPOR        -------------------MASSAVDQPEG---VDE------TILTLRKVLVNES--EPLARRFRALFSLKYIACLQPPT-EKTLPAIQAIAAGFT-SSSALLKHELAYCLGQTRNPDAV
C8VBH9_EMENI      ----------------------MTTDNLNS---ADT------TVQTLRNVLTSET--EPLARRFRALFSLKHLACLQPPT-EKTLPAIQAIAAGFS-SASALLKHELAYCLGQTRNTDAL
DOHH_COCIM        -------------------MASTIADNADNNGNSKD------SVQYLRKVLTSES--SPLAQRFRALFSLKHLASSKPPT-EETLPAIEAIAAAFS-SPSALLKHELAYCLGQTRNLDTV
DOHH_CHAGB        -------------------MTAPTTTSPSSEATTTTTTTTLNTIATLHQSLTTET--TPLPVRFRALFSLKHVAATHPATSAESLAAIDAIAAGFA-SPSALLKHELAYCLGQTANGAAI
DOHH_NEUCR        -------------------MSA--------------------TIASLRESLCSET--TPLPIRFRALFSLKHLAVQNKGT-ADSLSAIDAIAAAFA-SPSALLKHELAYCLGQTGSDAAI
DOHH_GIBZE        -------------------MSPSADTPEISNSADS-------TVLSLKKSLCSED--SPLPIRFRALFSLKHVATTADDD-ATRVAAIEAIAAGFA-SPSALLKHELAYCLGQTGNTAAV
B6K221_SCHJY      ----------------------MSSETANQKVDQKV-------IDELERVLVNADKKTHLSLRYRALFSLNALGKK------GDSRAIDAVYSAFG-DDSELLKHEMAYVLGQSGQKYAV
DOHH_SCHPO        ----------------------MSSE----PVPQAV-------IDELERVLVNLDKSNPLSFRYRALFSLNALAKK------GDKRAVDAIYKAFI-DDSELLKHEMAYVMGQSGQQYAV
B9WC15_CANDC      ------------------------MSTDEVNIDAAS-------LEELRDILINKTGDTKLANRYRALFNLKSVGSEHSDK-EKAHKAIKYIAECFN-DESELLKHEVAYVLGQTKDLYAA
DOHH_DEBHA        -------------------------MTEEINIDTAT-------LEQLRDVLTNKSGDVKLANRFRALFNLKCVGAESENQ-DEVHKAIDYIAESFK-DDSELLKHEVAYVLGQTKNLHAA
C4R113_PICPG      --------------------------MSDVN-EATS-------LERLRDVLINNTGDIKLAHRFRALFFLKSIGAEYNEKPEDANKAIKYIGECFA-DSSELLKHEVAYVLGQTKNMTSA
DOHH_ASHGO        -------------------MSTEFEKKMEEHIDSCS-------LEQLRDILVNKNGDAKLTNRFRALFNLKCVAEEFAQRPEEAQRAVEYICEAFA-DSSELLKHEVAYVLGQTGNLACA
DOHH_KLULA        -------------------MSTNFEKHFEVDVDNCS-------LEQLRDILVNNSGKAPLANRFRALFNLKGHAEEFASKPEDALKATQYLAEAFG-DESELLKHEVAYVLGQTKNMAGA
DOHH_CANGA        -------------------MSTNFEKHFEENVDDCN-------LEQLRDILVNKEGKSALANRFRALFNLKTAASEFEANPSDAEKAVQYMGETFG-DNSELLKHEVAYVLGQTKNLKAA
DOHH_YEAST        -------------------MSTNFEKHFQENVDECT-------LEQLRDILVNKSGKTVLANRFRALFNLKTVAEEFATKPEEAKKAIEYIAESFVNDKSELLKHEVAYVLGQTKNLDAA
DOHH_YARLI        -------------------------------MATET-------LPELKKVLLNEDGQTALALRFRALFSLKDMGEK------GDNGAIDVIAEGFK-DDSELLKHELAYVLGQTKNFHAV
DOHH_CRYNE        ---------------------------MSVQVSPEQ-------MATLKATLLNTPGNVPLHERFRALFMLKAVGG---------DEVVDIVSEGLK-DPSPLLKHELAYVLGQLLNTRAL
DOHH_LENED        -----------------------------MSLSATQ-------LKALEDSVLNTSGKVLLHDRVRALFTLKSLKN---------EDAIRIISKGFQ-DSAALLKHELAYCLGQIRNPLAL
DOHH_USTMA        -------------------------------------------MDALEKSLCDFTGSTPLDQRFRSLFTIKGLAATSDQH---MQRAISIISQAFS-DDSALLKHELAYVLGQLEDARAL
DOHH1_ORYSJ       -------------------------MESAEVAASSTFGPTPEMEKFLCDLLLDTA--QPIAERFRALFSLRNLHGD---------GPRCALLQAAR-DSSNLLAHEAAFALGQMQDAEAI
DOHH_ARATH        -------------------------MESNGSVSSMVN-----LEKFLCERLVDQS--QPISERFRALFSLRNLKGP---------GPRNALILASR-DSSNLLAHEAAFALGQMQDAEAI
DOHH_DICDI        ---------------------------MVVVTEEIVNG--------LKETLTDVS--QPIAKRFRSLFTLRNLNGP---------LCIDAMASALN-DKSALLRHEIAYCLGQMEDEYAL
C1BQB5_9MAXI      -----------------------------------------MELEKIGGVLRDPS--RPLKERFRAVFTLRGLGGK---------TALDIMLKTIQEDTSELLKHEIAYVMGQMQDPAAI
C1BVM4_9MAXI      -----------------------------------------MDIDRVGKVLHDKS--RPLKERFRALFTLRSIGGK---------SSLEWMQKTIKEDSSELLKHEIAYVMGQMQDAEAI
A4I2C0_LEIIN      --------------------------MSALNSRTVE------EVRKDYAKLLDPQ--EPLDSRMRELYRLKEDCLKTAAG------VTVILET-IDTTDSVLLQHELAYNAGQSGREEAV
L.donovani        --------------------------MSALNSRTVE------EVRKDYAKLLDPQ--EPLDSRMRELYRLKEDCLKTAAG------VTVILET-IDTTDSVLLQHELAYNAGQSGREEAV
Q4Q901_LEIMA      --------------------------MSASNSCTVE------EVRKEYAKLLDPQ--EPLDSRMRELYRLKEDCLKTVAG------VTVILEA-IDTTDSVLLQHELAYNAGQSGREEAV
Q38FR2_9TRYP      ------------------------------MDQDLS------VCEQEYRKLLDPE--EPLFSRTRELYRLKESILRTPAG------VHVLAKA-VDTTNSVLLQHELVYNLGQSAMVEAC
D0NC43_PHYIN      -----------------MPSHSSATETISTVVDTTP------SFEQLRDALLDLS--EPTGKRTRAIFYLRS--RGGLED------LQVLLTALLNRKDSELMRHELAYVIGQFQMEEAC
DOHH_ENCCU        -----------------------------------------MDIEVARKNIGCDS--VSIAKRMRSLFYLRNVLLP---------ESARAITEAFG-SKSVLLKHEAAYVLGQMRMEESV
C9QNK6_PLAFO      MGENNDNINIINNVNNNYNINSDNVGGDSSNKVRLIKYEESTNKEFILKYLVNIKN-DYIEKQMRALYECREVYKD------DIDEVINILRYALKNNDSVLLRHEIAYVIGQISNEKCN
                                                                  :           : * ::  .                            : *: ** .:  **
```

Figure S1

```
B0S4Z5_DANRE     PTLEAVLKDT-NQE--PMVRHEAGEALGAIG-NPKVLELLKKYAED---PVIEVAETCQLA---------------------VKRLEWLMNGGEQTKDG---------------TDENP
C1BJA2_OSMMO     PTLEAVLKDT-TQD--PMVRHEAGEALGAIG-NPKVLDLLKEYSED---PVVEVAETCQLA---------------------VKRLEWLMGGGEKKEDGA--------------TDGNP
DOHH_BOVIN       PVLLDVLRDT-RQE--PMVRHEAGEALGAIG-DPEVLEILKQYSTD---PVVEVAETCQLA---------------------VRRLEWLQQHGGESAVR------------------GP
DOHH_HUMAN       PMLVDVLQDT-RQE--PMVRHEAGEALGAIG-DPEVLEILKQYSSD---PVIEVAETCQLA---------------------VRRLEWLQQHGGEPAA-------------------GP
DOHH_MOUSE       PVLADVLQDT-SQE--PMVRHEAGEALGAIG-NPEVLGLLKQYSTD---PVVEVAETCQLA---------------------VGRLEWLQQHPGEATCA------------------GP
DOHH_CHICK       PALLGVLQDS-QQE--PMVRHEAGEALGAIG-DPEVLDVLRRYSED---PVVEVAETCQLA---------------------VRRLEWLQEHGEEPGS-------------------SP
DOHH_XENLA       PVLKQVLQDR-QQE--PMVRHEAGEALGAIG-DPEVLELLREYAQD---PVIEVAETCQLA---------------------VSRIEWLQKNPDSPDT-------------------NP
B0W942_CULQU     PILAKVLEDV-KQE--PMVRHEAAEALGAIG-ASEVEDILVKYSKD---PVVEVAETCEIA---------------------LGRVRWLQNQEQGFVDN------------------NP
DOHH_DROME       DILTKVLKDT-TQE--PMVRHEAAEAMGAIG-HPDVLPILEEYKQD---PVVEVAETCAIA---------------------LDRVRWLQSG-QKVDDS------------------NP
DOHH_CAEEL       PTLVSVLEDE-KQE--PMVRHEAGEALGAIA-DPSVKDVLRKYAQD---PCPEVSETCQIA---------------------LGRVEWVEKS--GKDTN------------------SP
B2WFV6_PYRTR     APLRGVLEDK-EED--SMCRHEAAEALGALS-DKSSLDLLRALRDDA-NEVDVVRETCDIA---------------------VERIEWDHGLQKGQ-------EKLKK---------SD
DOHH_PHANO       APLRGVLEDK-DED--SMCRHEAAEALGALS-DKGSLELLKKMRDDA-NEVDVVRETCDIA---------------------VDRIEWEHGLQKGT-------EKLKK---------SD
C0SAR9_PARBP     PYLREVLEDR-NED--AMCRHEAAEALGALG-DTSSLGTLRSLRDDE-NEVDVVRETCDLA---------------------VDRILWETSDQRKT-------EKLKK---------SD
C5K274_AJEDS     PYLREVLENR-AED--PMCRHEAAEALAALC-DSDSLDILRSFRDDE-NEPDVVRETCDIA---------------------VERIEWETSDRRKT-------ENLKQ---------SD
C5FM15_NANOT     PSLRHVLEDK-DED--SMCRHEAAEAIGALA-DTSSLDLLRKLRDDP-SEPEVVRETCEIA---------------------VDRILWETSEQRQT-------EKVKK---------SD
DOHH_ASPCL       PYLQEVVKDT-EQD--TMCRHEAAEALGALG-YEDSLEILKVLRDNK-DEPDVIRETCDIA---------------------VDRILWENSEQRKA-------EKLKA---------SD
DOHH_ASPFU       PFLQQVAKDT-EQD--TMCRHEAAEALGALG-YEDSLEILKALRDNQ-NEPEVIRETCDIA---------------------VDRILWENSEARKA-------EKLKT---------SD
DOHH_ASPNC       AFLQQVLKDK-EED--VMCRHEAAEALGALG-YEDSLEILKALKDDE-NEPEVIRETCDIA---------------------VDRIVWENSEARKA-------EKLKP---------SD
DOHH_ASPOR       SYLLEVVKNT-EQD--AMCRHEAAEGLGALG-FDTSLDVLKALRDDE-KEEDVIRETCDIA---------------------VDRILWENSEERKS-------EKLKP---------SD
C8VBH9_EMENI     PFLLDVVQDT-QED--SMCRHEAAEALGALG-YESSLEVLKALRDNE-NEVDVVRETCDIA---------------------VDRILWEQSEARKA-------EKLKP---------SD
DOHH_COCIM       PHLRKVLEDT-QED--AMCRHEAAEALGALG-DAGSLAILQRLRDDE-SEEEVVRETCDIA---------------------VDRILWETSKDSKS-------EKLKQ---------SD
DOHH_CHAGB       PYLTAVLEDT-GED--AMCRHEAAEALGALG-DVASLGVLKRFRDRE-GEEVVVTETCELA---------------------VERIEWENGEGKKA-------EKLRA---------SD
DOHH_NEUCR       PHLTQVLEDL-QED--PMCRHEAAEALGALG-KAESLGVLQKYLHRE-GEDVSVKETCEIA---------------------IDRIEWENSEERKQ-------EKLRQ---------SD
DOHH_GIBZE       KPLRQVLSDL-KED--PMCRHEAAEALGALG-WADNLDILREYRDRK-EEDISIVETCEIA---------------------IERIEWENSAERQK-------EKLRP---------SD
B6K221_SCHJY     EPLTKVLNDL-NQQ--VMVRHEAAEALGALG-FPEALPVLQKYYKE--DPLVPIKETCDLA---------------------INRICWKNGLERKN-------EVIST---------SE
DOHH_SCHPO       QPLINIVNDL-DQQ--VMVRHEAAEALGALG-FTESLPVLEKYYKE--DPLAPIRETCELA---------------------IARIQWKNGLDKNN-------EKITP---------SM
B9WC15_CANDC     PFLREVLEND-NQQ--CMVRHEAAEALGALG-DKESLPLLEKYFKD--DPSLEIRQTCELA---------------------IERIHWENSEKAKS-------EVLEK---------SL
DOHH_DEBHA       QYLRSVLENN-NQQ--IMVRHEAAEALGALG-DKDSLALLEDYFKN--DPSIEIKQTCELA---------------------IERIRWENSEKAKA-------ENLET---------SL
C4R113_PICPG     PILRDVLESK-EQQ--VMVRHEAAEALGALG-DKDSLDLLVKYYEDP-AEVEEIRQTCELA---------------------IERIKWENSSKAKE-------EKLET---------SL
DOHH_ASHGO       ATLREVMLDH-AQQ--CMVRHEASEALGALG-DAASLGALERSRRE--DPSEEVRQTSELA---------------------IERIRWQASGAAAT-------EQLQQ---------SL
DOHH_KLULA       PLLRDVLADD-KQQ--CMVRHEAAEALGALN-DVDSLDILEKYFKE--DPLLEIRQTCELA---------------------IDRIKWETSEEGRR-------EALQE---------SL
DOHH_CANGA       PLLRKTMLDL-AQQ--PMVRHEAAEALGALG-DKDSLEDLEKCLKN--DPHVAVRETCELA---------------------IARINWQHSDAPTK-------ESLQQ---------SL
DOHH_YEAST       PTLRHVMLDQ-NQE--PMVRHEAAEALGALG-DKDSLDDLNKAAKE--DPHVAVRETCELA---------------------INRINWTHGGAKDK-------ENLQQ---------SL
DOHH_YARLI       KPLQGVLADT-NQQ--AMVRHEAAEALGALG-DKGSVAMLQEYFEN--DPLEVIRETCELA---------------------LERIKWENSEAAKT-------ETLQK---------SA
DOHH_CRYNE       PTLSRVLENP-TGEHCSMVRHEAAEALGAIG-AEESLPILRKYMQD---ENREVRETCEIA---------------------VGKIEFDLSEEGKK-------TNANP----------D
DOHH_LENED       PVLESVLRNP-SED--PMVRHEAAEAMGAIS-TADSIPILKQYLSD---PDRSVRETCEIA---------------------IAKIEWDKTEEGAKNDKATRDENRLP----------L
DOHH_USTMA       PTLKKILQDL-SQD--AMVRHEAAEAMGAIS-DPSVLPILEQYRSD---SDVSVRETCELA---------------------ISKISFDNSEEGQALKQSKAQAKLAEEQSGLGGVESA
DOHH1_ORYSJ      PALEAVLKDL-SLH--PIVRHEAAEALGAIG-LEKSIPLLEESLAA--DPAVEVQETCELA---------------------LRRIEQQKNAGV--------SESTTI---------SP
DOHH_ARATH       PALESVLNDM-SLH--PIVRHEAAEALGAIG-LAGNVNILKKSLSS--DPAQEVRETCELA---------------------LKRIEDMSNVDAENQ-----SSTTEK---------SP
DOHH_DICDI       KVLIDLVKNS-DEH--PMVRHEAAEALGAIG-SESAHKTLKE-YSN--DPVREVSETCQLA---------------------LSRVEWYEKNKP---------ETEED---------KM
C1BQB5_9MAXI     EGLIDIPNGP-KME--PIVRHEAAEALGALGKDDNIRASLAKALASK-ELPVEVEETCSLA---------------------LRKLEWSKSPEENLSA-------------------NP
C1BVM4_9MAXI     EGLISILSDP-KME--PIVRHEAAEALGALGEDPKIRKALESILNSK-DLPIEVTETVSLA---------------------LSRLDWLKNPEKNLSK-------------------NP
A4I2C0_LEIIN     PELERILRTT-SYD--VVTRHEAAEALGAIG-SPLALQVLEAHSDPTTEPEAPIRETCELA---------------------LARIAMKETKGDAAVAP------PSG---------CE
L.donovani       PELERILRTT-SYD--VVTRHEAAEALGAIG-SPLALQVLEAHSDPTTEPEAPIRETCELA---------------------LARIAMKETKGDAAVAP------PSG---------CE
Q4Q901_LEIMA     PELERILRTT-SYD--VVTRHEAAEALGAIG-SPLALQVLETHSAPTTEPEASIRETCELA---------------------LARIAMKETKGDAAVAP------PSG---------CE
Q38FR2_9TRYP     PHLERFIRAVGKYD--IVTRHEAVEALGAIG-DPACIPLLRHFMEPANEPEAAIRESCELA---------------------LKRIEMLEEKGKEAVGP------AAN---------CP
D0NC43_PHYIN     ETLQQVLADE-ADD--GMVRHEAAEALGAIG-ASQSLPVLEKYSA---DPAPEVSDTCKLAVT-------------------LVKYKLAKAKGEVVEGE------VDR---------NP
DOHH_ENCCU       RVLLDVLSDE-DED--EIVRHEAGEALGNFRPREEIVEALRKYSNH---PKKPISETCYLA-----------------------LMKLKDGSDIVS----------------------K
C9QNK6_PLAFO     NILINLLSDE-NEN--IMVRHEAAKGLAAIG-SESNIPVIKKYLNDS---SVEVRETCELALSSLIEKNKYAACSCINKTVPYKNNVLNDNPKRDSSNNSNNNNNNNINNNNSNSHSNS
                   *          .   : **** :.:. :         :             : ::  :*
```

```
B0S4Z5_DANRE    YCS-VDPAPP-----AQRK-----------------SVPELRTQLLDETL--PLFDRYRAMFALRNLGTE-------EAVLALGDGLQ-C-SSALFRHEIGYVLGQIQHAASIPQLQAAL
C1BJA2_OSMMO    YFS-VDPAPP-----ASNQ-----------------DVADLRRQLLDESL--PLFERYRAMFALRNLGTK-------EAVLALGDGLQ-C-GSALFRHEIAYVLGQVQHEASVPQLRAAL
DOHH_BOVIN      YLS-VDPAPP-----AEER-----------------DLGQLREALLDEAR--PLFDRYRAMFALRDAGGK-------EALALAEGLR-C-GSALFRHEIGYVLGQMQHEAAVPQLAAAL
DOHH_HUMAN      YLS-VDPAPP-----AEER-----------------DVGRLREALLDESR--PLFERYRAMFALRNAGGE-------EALALAEGLH-C-GSALFRHEVGYVLGQLQHEAAVPQLAAAL
DOHH_MOUSE      YLS-VDPAPP-----AAEQ-----------------DVGRLREALLDEAR--PLFERYRAMFALRNVGGK-------EALALAEGLQ-C-GSALFRHEVGYVLGQLQHEAAVPGLAATL
DOHH_CHICK      YRS-VDPAPP-----AEET-----------------DVATLRAVLLDESR--PLFDRYRAMFALRNLGGR-------DAVLALADGLR-A-GSALFRHEIGYVLGQMQDEACVPQLTAAL
DOHH_XENLA      YLS-VDPAPP-----AEEK-----------------DVPTLRATLLDETC--PLFHRYRAMFALRNIGGE-------EAVLALADGLQ-I-GGSLFRHEIGYVLGQMQHKAAVPGLSAAL
B0W942_CULQU    YAS-VDPTPP-----AVTN-----------------SVEELQRTLLDETD--SLFNRYRAMFSLRNLRTQ-------ESVLALASGLK-G-KSALFRHEVAFVLGQLQEECSVPFLAENL
DOHH_DROME      YAS-VDPSPP-----TAGDK----------------SVTELKAIYLDAQQ--SLFDRYRAMFSLRNLRTE-------ESVLAIAEGLK-D-SSALFRHEVAFVLGQLQEPCSIPFLQENL
DOHH_CAEEL      YDS-VDPTPS-----ASTS-----------------DVEELAATLIDASL--PLFDRYRAMFSLRNIKTD-------KSIKALAQGLYCE-DSALFRHEVAYVLGQLQSPVATQELKDRL
B2WFV6_PYRTR    FTS-IDPAPPLPQG-AEKPS-----------------IAELEKTLLDTAL--PLFQRYRAMFALRDLSSPPDLPTAVPAVQALARGFGD--PSALFRHEIAFVFGQLSHPASIPSLTEAL
DOHH_PHANO      FTS-VDPAPPMPES-NEAPS-----------------IPALEKTLLDTTL--PLFQRYRAMFALRDLSSPPDLPTAVPAVHALARGFGD--PSALFRHEIAFVFGQLSHPASIPSLTEAL
C0SAR9_PARBP    FAS-IDPAPPLPMP-TDEPS-----------------IPELKQTLLDSKL--PLFQRYRAMFALRDLASPPNLPTAVPAIEALAEGFKD--SSALFRHEIAFVFGQLSHPASIPSLTATL
C5K274_AJEDS    FAS-IDPAPPLPMP-ADEPS-----------------IPDLEKTLLDPKL--PLFQRYRAMFALRDLASPPDLPTAVPAVHALAEGFKD--SSALFRHEIAFVFGQLAHPASIPSLTATL
C5FM15_NANOT    FTS-IDPAPPTALT-TEEHS-----------------IPELKKTLLDTSI--PLFQRYRAMFALRDLSSPPDRPTAREAVEALAEGLKD--KSALFRHEIAFVFGQLSHPASIPSLVEAL
DOHH_ASPCL      FTS-IDPAPPLPMA-TSEPS-----------------IPDIEKRLLDTSL--PLFQRYRAMFALRDLASPPDLPTATHAVEALAKGLKD--PSALFRHEIAFVFGQLSHPASIPSLTEAL
DOHH_ASPFU      FTS-IDPAPPLPMT-ASEPS-----------------IPEIEQTLLDTSL--PLFLRYRAMFALRDLASPPDLPTATRAVEALAKGLKD--PSALFRHEIAFVFGQLCHPASIPSLTEAL
DOHH_ASPNC      FTS-IDPAPPMPLE-AAEPS-----------------IPELEKTLLDTKL--PLFQRYRAMFALRDLASPPDLPTAVQAVDALAKGLKD--PSALFRHEVAFVFGQLCHPASVPSLTECL
DOHH_ASPOR      FTS-IDPAPPLPMA-SSQPS-----------------ISDLEKTLLDTKL--PLFQRYRAMFALRDLASPPDLPTAVEAVEALAKGLKD--PSALFRHEVAFVFGQLCHPASVPSLTETL
C8VBH9_EMENI    FTS-IDPAPPMPLT-AKEPS-----------------IPDLEKTLLDTNL--PLFERYRAMFGLRDLASPPDLPTAKQAVQSLAKGMKD--PSALFRHEIAFVFGQLCHPASVPSLTETL
DOHH_COCIM      FTS-IDPAPPLPLS-SAEQS-----------------IPELKQILLDASL--PLFKRYRAMFALRDMCSPPDLPTAVPAIEALAEGFKD--RSALFRHEIAFVFGQLSHPASIPSLVATL
DOHH_CHAGB      FSS-VDPAPPTAQG-QEEQT-----------------VEELGNALMDTSL--PLFKRYRAMFALRDLASPPDLPTAVPAVLALAKGFAD--SSALFRHEIAFVFGQLAHPASIPALTEAL
DOHH_NEUCR      FAS-VDPAPPMPED-DEKQT-----------------VETLEKKLLDTSL--PLFKRYRAMFALRDLASPPDLPTAVPAILALAKGLKD--ESALFRHEIAFVFGQLSHPASIPALTEAL
DOHH_GIBZE      FAS-IDPAPPMPES-DKEAE-----------------VEDLGRKLMDTNA--DLFSRYRAMFALRDLASPPDLPTATPAVLALAKGLSD--SSALFRHEIAFVFGQLSHPASIPALTEAL
B6K221_SCHJY    YDDVIDPAPPLPDATAADVS---------------AEVKKLRSELIDQKL--PLFYRYRVMFRLRNIGTE-------EAVEALTDGFQD--PSALFKHEVAFVFGQLTSPKAIPSLVKVL
DOHH_SCHPO      YDSVVDPAPPMPDH-EQDVK---------------SEVAKLRSEIVDQNL--PLFYRYRVMFRLRNIGNE-------EAVLALTDGFKD--PSPLFRHEIAFVFGQMIAPASVPALIKVL
B9WC15_CANDC    YTS-IDPAPPLATN-DSTSK-----------------VEKLKEILNDQDK--PLFERYRAMFRLRDIGTD-------EACLALASGFDD--PSALFKHEIAYVFGQMCNPVTVPSLIKVL
DOHH_DEBHA      YTS-IDPAPPMPS--DQESK-----------------VEKLQKILNNQDE--PLFERYRAMFRLRDMGTD-------EACLALASGLDDD-PSALFKHEIAYVFGQLCNPVTVPALIKTL
C4R113_PICPG    YES-IDPAPPLAID-GSNTK-----------------ISKLQKILNDQNT--PLFERYRAMFRLRDIGTD-------EAALALATGFSD--PSALFKHEIAYVFGQLSNPVTVPSLVQVL
DOHH_ASHGO      YSS-VDPAPALSL--EKDYD-----------------VPQLQALLNDQRA--PLFERYRAMFRLRDIGSD-------EACYALASGFDD--PSALFKHEIAYVFGQIGNPCVVPHLQEVL
DOHH_KLULA      YSS-IDPAPPFSL--EKDYK-----------------IQELKDILNDQNR--PLFERYRAMFRLRDIGND-------EACLALASGFDD--PSALFKHEIAYVFGQICNPVVVPHLKEVL
DOHH_CANGA      YSS-IDPAPPLAL--EKEYD-----------------LEELKKLLNDQEK--PLFLRYRAMFRLRDIGTD-------EAVLALASGFND--PSALFKHEIAYVFGQMGSTAAVPSLTEVL
DOHH_YEAST      YSS-IDPAPPLPL--EKDAT-----------------IPELQALLNDPKQ--PLFQRYRAMFRLRDIGTD-------EAILALATGFSA--ESSLFKHEIAYVFGQIGSPAAVPSLIEVL
DOHH_YARLI      YTS-IDPAPPLPTD-QAEGSDK-------------ATVEKLQKTLMDKSQ--SLFHRYRAMFRLRDIGTE-------DAVLALATGFDD--SSALFRHEIAYVFGQMSDPASVPALIKVL
DOHH_CRYNE      FPT-IDPAPS------AAP----------------SDIPSLRADLLNTSL--PLFQRYRAMFALRDFGAG-----SKEAVEALADGFRD--GSALFRHEIAYIFGQLSSPYSIPSLLSRL
DOHH_LENED      YTS-IDPAPATSGLLTGAPRPEEIS---------QTKIDELRDNLLDVNR--PLFERYRAMFALRNIG-------SPAAVDALAAGFSG--DSALFKHEIAFVFGQLLSPHSVPCLIEVL
DOHH_USTMA      FKP-IDPAPAMTPAASKEAARSQADGVRYD----ASHVPLFQSTLLDTNL--SLFERYRAMFALRNVAHGGG-DGAIQAVLALARGLQD--GSALFRHEICFVFGELCHPASIPSMLHVL
DOHH1_ORYSJ     FLS-VDPALP-----AKQG----------------LSVHQLREILLNEQE--SMYERYAALFALRNDSRD-------AAVSAIVAALG-A-KSALLKHEVAYVLGQLQNKAASDALSTVL
DOHH_ARATH      FMS-VDPAGP-----AASF----------------SSVHQLRQVLLDETK--GMYERYAALFALRNHGGE-------EAVSAIVDSLS-A-SSALLRHEVAYVLGQLQSKTALATLSKVL
DOHH_DICDI      YMS-VDPAPP-----LKKGS---------------VSRDELRSKFLDSNL--DIFNRYRALFSLRDIGDE-------QSVLALCDGLKDQ-SSALLRHEVAFVLGQLQHRVAIDPLTTCV
C1BQB5_9MAXI    YDS-VDPAPPFP----KEYS------------------SDLGALLLNESE--DLFLRYRAMFSLRNKGDE-------ESVLALCKSLTEDKSSALFRHEVAYVLGQIQSPSSQKALLQSL
C1BVM4_9MAXI    YDS-VDPAPPFP----LDFS------------------SNLESLLHNESE--DIFIRYRAMFSLRNKGDT-------DSIQSLCRGLIHDKSSALFRHEVAYVLGQIQSFESKKSLIQIL
A4I2C0_LEIIN    FVS-VDPSPAFSALYSSTDEPV------------PHTVEELEAVLLDTSGHTRLFRRYMAMFTLRNLATE-------AAVAALCRGLREDNVSALFRHEVAFVLGQLERPSSQPALIAAL
L.donovani      FVS-VDPSPAFSALYSSTDEPV------------PHTVEELEAVLLDTSGRTRLFRRYMAMFTLRNLATE-------AAVAALCRGLREDNVSALFRHEVAFVLGQLERPSSQPALIAAL
Q4Q901_LEIMA    FVS-VDPSPAFSALYSSTDEPV------------PLTVEELEAVLLDTSGRTRLFRRYMAMFTLRNLATE-------AAVAALCRGLREDTISALFRHEVAFVLGQLERPSSQPALIAAL
Q38FR2_9TRYP    FVS-IDPAPAFNG--TNTGGSA------------PYTVSELENLLCDTTGAVSLWLRYQAMFTLRNIGTP-------EAVAALSRALRQDNTSALLRHEVAFVLGQLEHPASQPALLDAL
D0NC43_PHYIN    YLS-EDPAPAAEK---------------------DVSTAELRKVLLDPNG--DMFARYRAMFRLRNRNTE-------EAALALAEAFQDP--NALFKHEVAYVMGQMENPVLVPALKKVL
DOHH_ENCCU      FGS-RDPALP------MEGS-----------------FEEARRILLDKNE--CLYRRYQAMFYLRDLGTS-------AAIHALGKSMED--DSALFKHEVSFVFGQMRSRESIPYLIKGM
C9QNK6_PLAFO    FSNSQDDADDDIYFHSKKKFNTIDPVVCISDSNNKKHVNDLIRDLNNEAL--ALKNRYEALFLLRDMETD-------TSLNALGEALVKDKSSAIFRHELAFVLGQVLHLNSLKYLLSSL
                :    * :                                       :      : ** .:* **:             :  ::  .:    ..:::**: :::*::         :   :
```

```
B0S4Z5_DANRE      EKMDENAMVRHECAEALGSIGKE--------------------PCVQILERYRK--DQERVVKESCEVALDMLEYENSSQFQYADGL----------------------LRLQSAH-----
C1BJA2_OSMMO      ERQDESPMVRHECAEALGSIGRQ--------------------ECMEVLETHRR--DQERVVKESCEVALDMLRYENSGQFQYADGL----------------------ARL---------
DOHH_BOVIN        AQPTENPMVRHECAEALGAIARP--------------------ACLAALRAHVA--DPERVVRESCEVALDMYEYETGSTFQYADGL----------------------ERLRSPLS----
DOHH_HUMAN        ARCTENPMVRHECAEALGAIARP--------------------ACLAALLQAHAD--DPERVVRESCEVALDMYEHETGRAFQYADGL----------------------EQLRGAPS----
DOHH_MOUSE        ARTTESPMVRHECAEALGAIARP--------------------ACLAALREHIE--DPEQVVRESCEVALDMYEYESSQDFQYADGL----------------------ERLRPPP-----
DOHH_CHICK        RSRAENPMVRHECAEALGSIARP--------------------SCLETLRAFAQ--DEERVVRESCEVALDMYEYENGPQFQYADGL----------------------CRLQA-------
DOHH_XENLA        ERFEENPMVRHECAEALGSIAHE--------------------DCLKALRAHVG--DGERVVRESCEVALDMHDYENSGDFQYANGL----------------------SQICEQI-----
B0W942_CULQU      RDASENEMVRHECAEALGAIATE--------------------ECTKILNEYLA--DEKRVVKESCEVALDMCEYENSPEFQYADTL----------------------TKVSH-------
DOHH_DROME        EDRLENEMVRHECAEALGAIATE--------------------DCIQILNRYAE--DDKRVVKESCVIALDMCEYENSPEFQYADGL----------------------AKLDATK-----
DOHH_CAEEL        LLSTENCMVRHECAEALGAIANE--------------------ECTEILKQYVN--DEERVVRESCEVALDMAEYENSDDLQYAHV-----------------------------------
B2WFV6_PYRTR      SNTNEASMVRHEAAEALGSLGDE-------------------DGVEDTLRKFLN--DPEQVVRDSVVVALDMAEFEKNGEVEYAIVP----------------------QAVAA-------
DOHH_PHANO        SNTKEASMVRHEAAEALGSLGDE-------------------EGVEETLKKFLN--DPEQVVRDSVIVALDMAEFEKNGEVEYAIVP----------------------QAQAIAA-----
C0SAR9_PARBP      NNREEASMVRHEAAEALGSLGEE-------------------EGVEETLRLFLN--DPEQVVRDSVVVALDMAEYEKNREKEYALLP----------------------QPATASA-----
C5K274_AJEDS      HDMKEASMVRHEAAEALGSLGDE-------------------EGVEETLKKFLD--DPEQVVRDSVIVALDMAEFEKNGEKEYALIP----------------------EAVATA------
C5FM15_NANOT      SNKEEASMVRHEAAEALGSLGDE-------------------EGVEEVLRKFLD--DPEVVVKDSVVVALDMAEYEKNGELEYSLIP----------------------EPVAAS------
DOHH_ASPCL        SDQNEVGMVRHEAAEALGSLGDC-------------------EGVEDTLKKFLN--DPEQVVRDSVIVALDMAEYEKNGEIEYALVP----------------------DSGVAAA-----
DOHH_ASPFU        SNQSEAGMVRHEAAEALGSLGDY-------------------EGVEETLRKFLN--DPEQVVRDSVIVALDMAEYEKNGEVEYALVP----------------------DAGVAAA-----
DOHH_ASPNC        SNQEEAGMVRHEAAEALGSLGDV-------------------EGVEDTLKKFLN--DPEQVVRDSIIVALDMAEFEKNGEMEYALVP----------------------DSGAPAAVSA--
DOHH_ASPOR        SDQKEMGMVRHEAAEALGSLGDV-------------------EGVEDTLKKFLN--DPEQVVRDSIIVALDMAEYEKNGEMEYALVP----------------------DSAAPAAVSAA-
C8VBH9_EMENI      SDLNEVGMVRHEAAEALGSLGDV-------------------EGVEDTLKKFLN--DPEKVVRDSIIVALDMAEFEKNGEIEYALIP----------------------DSGNPAAVPAA-
DOHH_COCIM        SDKNEVGMVRHEAAEALGSLGAE-------------------DGVEETLKRFVN--DPETVVRDSIIVALDMAEYEKSGEQEYILEQ----------------------PVAA--------
DOHH_CHAGB        SNTEEASMVRHEAAEALGSLGDE-------------------EGVEETLRKFLH--DAEAVVRESVIVALDMAEYEKSNETEYALIP----------------------EAQGTA------
DOHH_NEUCR        SNLDEVSMVRHEAAEALGSLGDE-------------------EGVEETLLKFLH--DKEKVVRESVIVALDMAEFEQSGQAEYALIP----------------------EVASKAS-----
DOHH_GIBZE        SNTNEASMVRHEAAEALGSLGEK-------------------DGVEDTLRKFLH--DKEKVVRESCIVALDIAEYEKGEDAEYALIP----------------------ESAGAAA-----
B6K221_SCHJY      EDKSEAPMVRHEAAEALGGIAT--------------------AECIPVLEKYAK--DDVRVVAESCIVALDMIKYEQSGEMEYAYVP----------------------KAGGKVPLENEE
DOHH_SCHPO        ENTEEVPMVRHEAAEALGGIAN--------------------DECLPVLKKFSK--DDVRVVAESCIVALDMIEYEKSGDMEYAYIP----------------------KVSA--------
B9WC15_CANDC      KDESQAGMVRHEAAEALGSIAT--------------------DECLPVLQSFLN--DSEPVVRDSAIVALDMYEYENSNELEYATVK----------------------------------
DOHH_DEBHA        KDEREAAMVRHEAAEALGSIAT--------------------DECLPVLQSFLN--DKDQVVRDSAVVALDMYEYENSTEI----------------------------------------
C4R113_PICPG      KDTSQAAMVRHEAAEALGSIAT--------------------DEVLPILQGFLK--DEDEVVRDSAVVALDMYEYENSNEVEYAAV-----------------------------------
DOHH_ASHGO        KREHEAPMVRHEAAEALGSIAT--------------------DDVLPVLKRHLQ--DKDEVVRESAAIALDMYDYENSNELEYAPA-----------------------------------
DOHH_KLULA        ARPEEAPMVRHEAAEALGSIAT--------------------DDVLPVLKEHLK--DSDSVVRESAIVALDMYEYENSNDLEYAPV-----------------------------------
DOHH_CANGA        GRKEEAPMVRHEAAEALGAIAS--------------------EDALPILKQYLN--DEVDVVRESAIVALDMWEYENSNELEYAPA-----------------------------------
DOHH_YEAST        GRKEEAPMVRHEAAEALGAIAS--------------------PEVVDVLKSYLN--DEVDVVRESCIVALDMYDYENSNELEYAPTA----------------------N-----------
DOHH_YARLI        GKTEEEGMVRHEAAEALGSIAT--------------------DDVLPILKKFAE--DKDQVVRESAIVALDMYEYENSNEVEYAH------------------------------------
DOHH_CRYNE        RDAKEDDMVRHEAAEALGGIASDGVESENPEVVLPEDERLPEGGVLAVLREWAVKADAPTVVRESCQVAIDMWEYENSADQFNPLDSLSAKQEEREKTEKVNTTGMERSAHAAVAAMGIAA
DOHH_LENED        QNSPESDMVRHEAAEALGGIAT-------PEVLPP-------------LKEWVARDDAPVVVRESCQVALDLWEYENSGD-FQYAN------------------GLE-SPSTPISV-----
DOHH_USTMA        NDTKEHEMVRHEAAEALGGIVEEGEHAANDSANDYTR-------VLDTLNKWAHDMDAPRVVRESCIVALDELAYNNDPTQFHRIE-----------------------TSTPVAA-----
DOHH1_ORYSJ       KNVDEHPMVRHEAAEALGSIADQ--------------------ESIALLEEFAK--DPEPIVSQSCEVALSMLEYERSGKSFEF----------------------LFLQTPGVQQES---
DOHH_ARATH        RDVNEHPMVRHEAAEALGSIADE--------------------QSIALLEEFSK--DPEPIVAQSCEVALSMLEFENSGKSFEF----------------------FFTQDPLVH------
DOHH_DICDI        LDESENAMVRHEAAEALGAIAST--------------------ETIPLLEKLLQ--DKEPIVSESCAVALDVTEYFNNTESFQYADGI-----------------KILLEKNLVDQQQK--
C1BQB5_9MAXI      HNETEHPMVRHEAAEALGSLGTP--------------------EIHEELKKFTG-PKVPQVVRQSCEVALDFVDYNQSGEFQYANTLN---------------------LVQSCS------
C1BVM4_9MAXI      QNEKEHPMVRHECAEALGSIGTP--------------------EIHSELQKYLG-KEVPAVVRESCEIALDFCDYNQSSDFQYANTLS---------------------SSIKS-------
A4I2C0_LEIIN      KDEEEAPMVRHEAAEALGAIADP--------------------ATLPVLESYAT--HHEPIVRDSCVVALEMHKYWANFNSLAHQQQQEA-------------------------------
L.donovani        KDEEEAPMVRHEAAEALGAIADP--------------------ATLPVLESYAT--HHEPIVRDSCVVALEMHKYWANFNGLAHQQQQEA-------------------------------
Q4Q901_LEIMA      KDEEEAPMVRHEAAEALGAIADP--------------------ATLPVLESYAT--HHEPIVRDSCVVALEMHKYWAHFNSLAHQQQEA--------------------------------
Q38FR2_9TRYP      RDEHEAPMVRHEAAEALGAIADP--------------------KTLPALEEYAK--HKEAIVRDSCVVALEMHKYWSQFNNQRIQH-----------------------------------
D0NC43_PHYIN      LDETEHRMVRHEAAEALGAIGST--------------------ECEKILKQYLK--DDVQVVRESCEVALDIMDYWAPTK-----------------------------------------
DOHH_ENCCU        EDEKEHGMVRHECAEALGAIGDD--------------------AALKALSKYLH--DPCDILRESVEVAVDIHSYMTGDEIEYCNAE----------------------------------
C9QNK6_PLAFO      TNVSEHEMVRHEVALALGSLGSLN--------LNSDEYKIIQEQIIDTLKKYSK--DECVVVAESCLVGLDYISENLNISIEVH-------------------------------------
                      :  ***** * ***.:                            *       .   :: :*  :.:.
```
