# Supplementary figures and images for: A Unique Modification of the Eukaryotic Initiation Factor 5A Shows the Presence of the Complete Hypusine Pathway in Leishmania donovani
Source: PLoS One. 2012 Mar 16;7(3):e33138. doi: 10.1371/journal.pone.0033138 (PMC3306375; doi:10.1371/journal.pone.0033138)

Figure S2

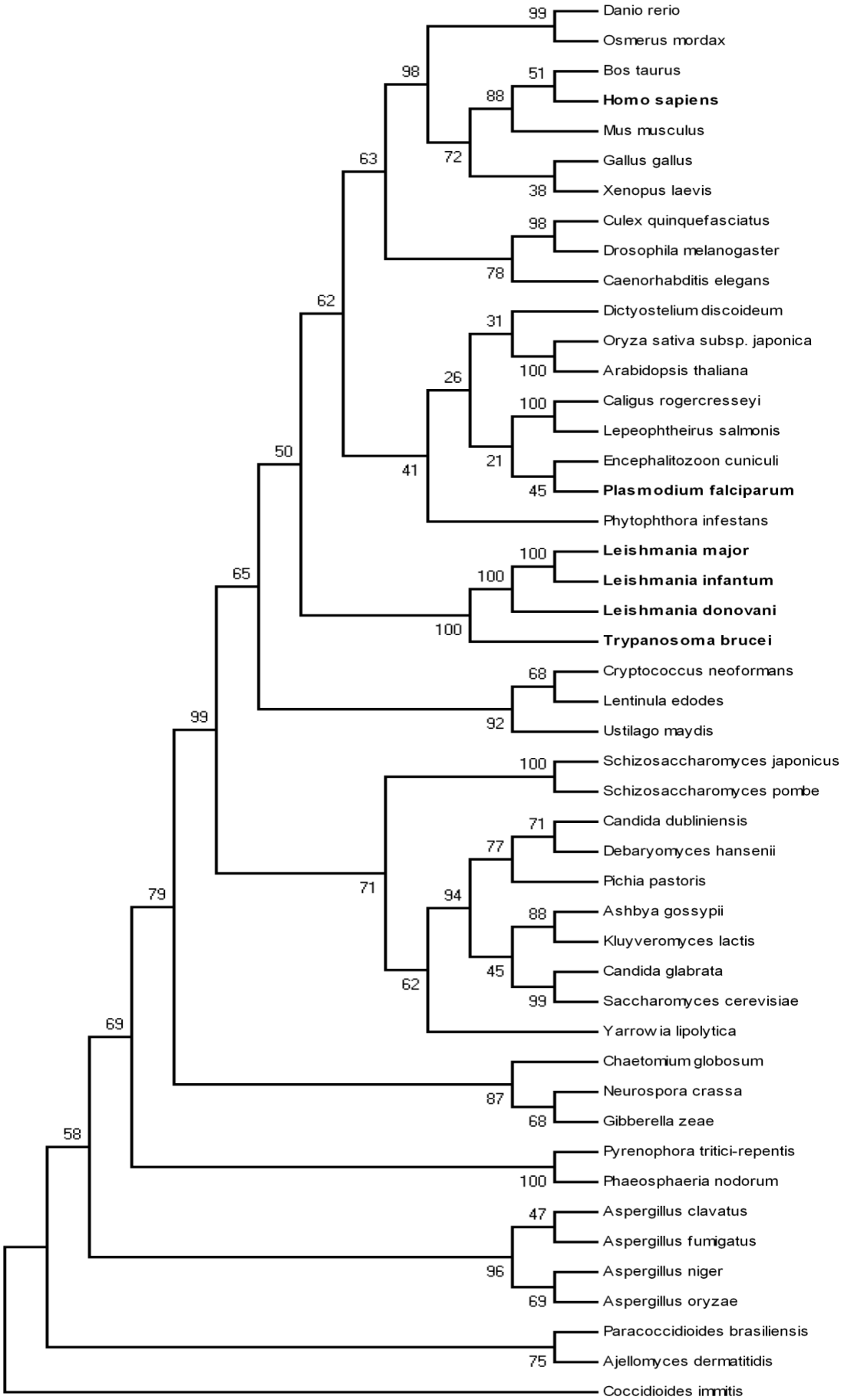

Supplement: Figure S2 — Phylogenetic analysis of DOHH protein sequences from different eukaryotic sources. The phylogram presented is a consensus of 1000 bootstrap replicates constructed using the MEGA program (Ver. 4.0). The numbers at the node present the percentage of trees with the same node among all the bootstraps. L. donovani DOHH protein sequence clusters with other eukaryotic pathogens such as Plasmodium falciparum and Trypanosoma brucei. (PDF) [file pone.0033138.s002.pdf]
