# Supplementary material for: A Unique Modification of the Eukaryotic Initiation Factor 5A Shows the Presence of the Complete Hypusine Pathway in Leishmania donovani
Source: PLoS One. 2012 Mar 16;7(3):e33138. doi: 10.1371/journal.pone.0033138 (PMC3306375; doi:10.1371/journal.pone.0033138)
Supplement: Information S1 — Amino Acid Conservation Scores. (PDF) [file pone.0033138.s003.pdf]

Supporting Information S1

Amino Acid Conservation Scores

===============================

- POS: The position of the AA in the SEQRES derived sequence.
- SEQ: The SEQRES derived sequence in one letter code.
- 3LATOM: The ATOM derived sequence in three letter code, including the AA's positions as they appear in the PDB file and the chain identifier.
- SCORE: The normalized conservation scores.
- COLOR: The color scale representing the conservation scores (9 - conserved, 1 - variable).
- CONFIDENCE INTERVAL: When using the bayesian method for calculating rates, a confidence interval is assigned to each of the inferred evolutionary conservation scores.
- CONFIDENCE INTERVAL COLORS: When using the bayesian method for calculating rates. The color scale representing the lower and upper bounds of the confidence interval.
- MSA DATA: The number of aligned sequences having an amino acid (non-gapped) from the overall number of sequences at each position.
- RESIDUE VARIETY: The residues variety at each position of the multiple sequence alignment.

POS	SEQ	3LATOM	SCORE (normalized)	COLOR	CONFIDENCE INTERVAL	CONFIDENCE INTERVAL COLORS	MSA DATA	RESIDUE VARIETY
1	M	MET1:	0.427	3*	-0.358, 0.912	6,1	3/50	M,N,S
2	S	SER2:	0.077	5*	-0.745, 0.396	8,3	3/50	E,S
3	A	ALA3:	0.613	3*	-0.172, 0.912	6,1	3/50	A,E,N
4	L	LEU4:	0.912	1*	0.068, 2.321	5,1	3/50	L,N,P
5	N	ASN5:	1.057	1*	0.068, 2.321	5,1	4/50	L,N,V
6	S	SER6:	1.056	1*	0.068, 2.321	5,1	4/50	E,G,P,S
7	R	ARG7:	1.279	1*	0.396, 2.321	3,1	5/50	G,N,P,Q,R
8	T	THR8:	0.118	5	-0.358, 0.396	6,3	10/50	A,E,S,T,V
9	V	VAL9:	0.749	2*	0.068, 0.912	5,1	15/50	E,K,Q,S,T,V
10	E	GLU10:	0.378	4*	-0.172, 0.912	6,1	19/50	A,D,E,I,L,M,Q
11	E	GLU11:	0.318	4*	-0.172, 0.912	6,1	19/50	A,D,E,K,P,Q,S,T
12	V	VAL12:	-0.110	5	-0.358, 0.068	6,5	19/50	A,E,I,Q,T,V
13	R	ARG13:	0.428	3*	-0.172, 0.912	6,1	19/50	A,D,E,L,Q,R,T
14	K	LYS14:	0.349	4*	-0.172, 0.912	6,1	19/50	A,E,K,R,S,T,Y
15	D	ASP15:	0.892	1	0.396, 0.912	3,1	19/50	A,D,I,K,L,R,V
16	Y	TYR16:	-0.073	5	-0.358, 0.068	6,5	19/50	G,I,R,S,T,V,Y
17	A	ALA17:	1.148	1	0.396, 2.321	3,1	20/50	A,E,G,K,L,Q,R,S,V
18	K	LYS18:	0.482	3*	0.068, 0.912	5,1	22/50	C,I,K,L,R,S,T,V
19	L	LEU19:	-0.664	8	-0.838,-0.510	8,7	34/50	D,L,N,V
20	L	LEU20:	1.277	1	0.912, 2.321	1,1	35/50	C,E,F,I,K,L,N,T,V
21	D	ASP21:	0.013	5	-0.172, 0.396	6,3	36/50	D,E,N,P,S,T
22	P	PRO22:	0.850	2	0.396, 0.912	3,1	36/50	A,E,G,K,P,Q,S,T,V
23	Q	GLN23:	1.823	1	0.912, 2.321	1,1	37/50	A,D,E,G,K,N,Q,S,T
24	E	GLU24:	0.320	4	0.068, 0.396	5,3	38/50	A,E,K,N,Q,R,S,T,V
25	P	PRO25:	-0.288	6	-0.510,-0.172	7,6	41/50	A,D,K,L,P,V
26	L	LEU26:	-0.975	9	-1.101,-0.919	9,9	45/50	I,L

27	D	ASP27:	0.100	5	-0.172, 0.396	6,3	45/50	A,D,G,H,K,P,Q,S,T
28	S	SER28:	0.607	3	0.396, 0.912	3,1	45/50	A,D,E,F,I,K,L,N,Q,R,S,T,V
29	R	ARG29:	-1.125	9	-1.176,-1.101	9,9	48/50	R
30	M	MET30:	-0.711	8	-0.919,-0.637	9,8	48/50	F,M,V,Y
31	R	ARG31:	-1.125	9	-1.176,-1.101	9,9	48/50	R
32	E	GLU32:	-0.841	8	-0.989,-0.745	9,8	48/50	A,E,S
33	L	LEU33:	-1.096	9	-1.176,-1.049	9,9	48/50	L
34	Y	TYR34:	-0.966	9	-1.101,-0.919	9,9	48/50	F,Y
35	R	ARG35:	-0.599	7	-0.745,-0.510	8,7	48/50	I,M,N,R,S,T,Y
36	L	LEU36:	-0.976	9	-1.101,-0.919	9,9	48/50	I,L
37	K	LYS37:	-0.748	8	-0.919,-0.637	9,8	48/50	K,N,R
38	E	GLU38:	0.174	4	-0.172, 0.396	6,3	48/50	A,C,D,E,G,H,N,S,T,Y
39	D	ASP39:	0.583	3	0.396, 0.912	3,1	48/50	A,D,H,I,L,M,V
40	C	CYS40:	0.249	4	0.068, 0.396	5,3	48/50	A,C,G,H,K,L,N,R
41	L	LEU41:	0.987	1	0.396, 0.912	3,1	48/50	A,C,E,G,K,L,N,S,T,V
42	K	LYS42:	2.088	1	2.321, 2.321	1,1	26/50	D,E,G,H,K,Q,R,T
43	T	THR43:	-0.015	5	-0.358, 0.396	6,3	26/50	A,D,M,R,S,T,V
44	A	ALA44:	2.150	1	2.321, 2.321	1,1	48/50	A,D,E,G,H,K,L,N,P,Q,V
45	A	ALA45:	2.053	1	2.321, 2.321	1,1	48/50	A,D,E,G,K,L,P,R,S,T,V
46	G	GLY46:	-0.651	8	-0.838,-0.510	8,7	48/50	A,C,G,P,S,V
47	V	VAL47:	-0.415	7	-0.637,-0.172	8,6	48/50	A,I,R,T,V
48	T	THR48:	1.898	1	0.912, 2.321	1,1	48/50	A,C,D,E,K,N,Q,R,S,T
49	V	VAL49:	-0.438	7	-0.637,-0.358	8,6	48/50	A,C,I,V,W,Y
50	I	ILE50:	-0.756	8	-0.919,-0.637	9,8	48/50	I,L,M,V
51	L	LEU51:	-0.087	5	-0.358, 0.068	6,5	48/50	A,G,I,L,S,T,V,Y
52	E	GLU52:	0.023	5	-0.172, 0.396	6,3	48/50	A,E,K,L,Q,R,S
53	T	THR53:	0.226	4	0.068, 0.396	5,3	48/50	A,C,G,S,T
54	I	ILE54:	-0.304	6	-0.510,-0.172	7,6	48/50	A,F,I,L,S
55	D	ASP55:	0.333	4*	-0.510, 0.912	7,1	3/50	D,V
56	T	THR56:	1.593	1	0.912, 2.321	1,1	48/50	A,D,E,G,I,K,N,Q,R,S,T,V
57	T	THR57:	-0.978	9	-1.049,-0.919	9,9	48/50	D,S,T
58	D	ASP58:	1.165	1	0.912, 2.321	1,1	49/50	A,D,E,K,N,P,S
59	S	SER59:	-1.086	9	-1.144,-1.049	9,9	50/50	A,S
60	V	VAL60:	-0.795	8	-0.919,-0.745	9,8	50/50	A,E,N,P,V
61	L	LEU61:	-1.097	9	-1.176,-1.049	9,9	50/50	L
62	L	LEU62:	-1.097	9	-1.176,-1.049	9,9	50/50	L
63	Q	GLN63:	-0.832	8	-0.989,-0.745	9,8	50/50	A,K,Q,R
64	H	HIS64:	-1.131	9	-1.176,-1.101	9,9	50/50	H
65	E	GLU65:	-1.121	9	-1.176,-1.101	9,9	50/50	E
66	L	LEU66:	-0.496	7	-0.745,-0.358	8,6	50/50	A,I,L,M,V
67	A	ALA67:	-1.136	9	-1.176,-1.101	9,9	50/50	A

68	Y	TYR68:	-0.829	8	-0.989,-0.745	9,8	50/50	F,Y
69	N	ASN69:	-0.426	7	-0.637,-0.172	8,6	50/50	A,C,N,V
70	A	ALA70:	-0.837	8	-0.989,-0.745	9,8	50/50	A,L,M
71	G	GLY71:	-1.095	9	-1.176,-1.049	9,9	50/50	G
72	Q	GLN72:	-1.127	9	-1.176,-1.101	9,9	50/50	Q
73	S	SER73:	-0.581	7	-0.745,-0.510	8,7	50/50	I,L,M,S,T
74	G	GLY74:	0.503	3*	0.068, 0.912	5,1	50/50	A,E,G,K,L,Q,R
75	R	ARG75:	-0.342	6	-0.510,-0.172	7,6	50/50	D,H,M,N,Q,R,S
76	E	GLU76:	2.219	1	2.321, 2.321	1,1	50/50	A,D,E,F,G,K,L,M,P,Q,R,T
77	E	GLU77:	1.760	1	0.912, 2.321	1,1	50/50	A,D,E,H,K,L,Q,R,S,T,Y
78	A	ALA78:	-0.776	8	-0.919,-0.637	9,8	50/50	A,C,G,S,T
79	V	VAL79:	-0.155	6	-0.358, 0.068	6,5	50/50	A,I,L,V
80	P	PRO80:	0.523	3*	0.068, 0.912	5,1	50/50	A,D,E,K,P,Q,R,S
81	E	GLU81:	2.114	1	2.321, 2.321	1,1	50/50	A,E,F,H,I,L,M,P,T,V,Y
82	L	LEU82:	-1.097	9	-1.176,-1.049	9,9	50/50	L
83	E	GLU83:	2.181	1	2.321, 2.321	1,1	50/50	A,E,I,K,L,Q,R,S,T,V
84	R	ARG84:	2.315	1	2.321, 2.321	1,1	50/50	A,D,E,G,H,K,N,Q,R,S
85	I	ILE85:	-0.770	8	-0.919,-0.637	9,8	50/50	I,L,T,V
86	L	LEU86:	-0.275	6	-0.510,-0.172	7,6	50/50	A,L,M,V
87	R	ARG87:	1.296	1	0.912, 2.321	1,1	50/50	A,E,K,L,N,Q,R,S
88	T	THR88:	-0.668	8	-0.838,-0.510	8,7	50/50	D,N,T
89	T	THR89:	1.427	1	0.912, 2.321	1,1	50/50	D,E,H,K,L,M,N,P,Q,R,S,T
90	S	SER90:	1.179	1	0.912, 2.321	1,1	50/50	A,D,E,G,K,N,Q,R,S,T
91	Y	TYR91:	-0.261	6	-0.510,-0.172	7,6	50/50	E,G,L,Q,Y
92	D	ASP92:	-0.684	8	-0.838,-0.510	8,7	50/50	D,E,H,Q
93	V	VAL93:	0.644	2	0.396, 0.912	3,1	50/50	A,C,E,I,P,S,T,V
94	V	VAL94:	-0.915	9	-0.989,-0.838	9,8	50/50	I,M,V
95	T	THR95:	-0.971	9	-1.049,-0.919	9,9	50/50	C,T,V
96	R	ARG96:	-1.126	9	-1.176,-1.101	9,9	50/50	R
97	H	HIS97:	-1.131	9	-1.176,-1.101	9,9	50/50	H
98	E	GLU98:	-1.121	9	-1.176,-1.101	9,9	50/50	E
99	A	ALA99:	-1.136	9	-1.176,-1.101	9,9	50/50	A
100	A	ALA100:	-0.903	9	-0.989,-0.838	9,8	50/50	A,G,S
101	E	GLU101:	-1.121	9	-1.176,-1.101	9,9	50/50	E
102	A	ALA102:	-1.071	9	-1.144,-1.049	9,9	50/50	A,G
103	L	LEU103:	-0.758	8	-0.919,-0.637	9,8	50/50	L,M
104	G	GLY104:	-1.095	9	-1.176,-1.049	9,9	50/50	G
105	A	ALA105:	-1.060	9	-1.144,-0.989	9,9	50/50	A,N
106	I	ILE106:	-0.853	8	-0.989,-0.745	9,8	50/50	F,I,L
107	G	GLY107:	-0.372	6	-0.637,-0.172	8,6	50/50	A,G,N,R,S
108	S	SER108:	0.791	2	0.396, 0.912	3,1	50/50	A,D,F,H,K,L,N,P,S,T,W,Y

109	P	PRO109:	1.087	1	0.396, 0.912	3,1	50/50	A,D,E,K,P,T,V
110	L	LEU110:	2.017	1	2.321, 2.321	1,1	50/50	A,D,E,G,K,L,S,T
111	A	ALA111:	-0.608	7	-0.745,-0.510	8,7	50/50	A,I,N,S,V
112	L	LEU112:	-0.172	6	-0.358, 0.068	6,5	50/50	H,I,K,L,V
113	Q	GLN113:	2.024	1	2.321, 2.321	1,1	50/50	A,D,E,G,K,N,P,Q,S
114	V	VAL114:	0.756	2	0.396, 0.912	3,1	50/50	A,D,I,L,M,T,V
115	L	LEU115:	-1.097	9	-1.176,-1.049	9,9	50/50	L
116	E	GLU116:	0.471	3*	0.068, 0.912	5,1	50/50	E,K,N,Q,R
117	A	ALA117:	0.809	2	0.396, 0.912	3,1	50/50	A,D,E,K,Q,R,V
118	H	HIS118:	-0.036	5	-0.358, 0.068	6,5	50/50	A,C,F,H,L,M,S,Y
119	S	SER119:	1.206	1	0.396, 2.321	3,1	29/50	A,F,K,L,R,S,Y
120	D	ASP120:	-0.660	8	-0.989,-0.510	9,7	12/50	D,H
121	P	PRO121:	0.824	2*	0.068, 2.321	5,1	4/50	D,N,P
122	T	THR122:	1.069	1	0.396, 0.912	3,1	50/50	A,D,E,K,L,M,N,Q,R,S,T
123	T	THR123:	2.257	1	2.321, 2.321	1,1	50/50	A,D,E,K,N,Q,S,T,V
124	E	GLU124:	-0.290	6	-0.510,-0.172	7,6	50/50	D,E,G,H,K,N,S
125	P	PRO125:	-0.384	7	-0.637,-0.172	8,6	50/50	E,P,S
126	E	GLU126:	0.570	3	0.396, 0.912	3,1	50/50	A,C,D,E,H,K,L,N,S,V
127	A	ALA127:	2.080	1	2.321, 2.321	1,1	50/50	A,D,E,I,K,L,P,Q,R,V
128	P	PRO128:	-0.136	6	-0.358, 0.068	6,5	47/50	A,E,P,S,V
129	I	ILE129:	-0.655	8	-0.838,-0.510	8,7	50/50	I,V
130	R	ARG130:	-0.293	6	-0.510,-0.172	7,6	50/50	A,K,Q,R,S,T,V
131	E	GLU131:	-0.944	9	-1.049,-0.838	9,8	50/50	E,Q
132	T	THR132:	-1.139	9	-1.176,-1.101	9,9	50/50	T
133	C	CYS133:	-0.951	9	-1.101,-0.838	9,8	50/50	C,S
134	E	GLU134:	-0.614	7	-0.745,-0.510	8,7	50/50	A,D,E,Q,Y
135	L	LEU135:	-0.533	7	-0.745,-0.358	8,6	50/50	I,L
136	A	ALA136:	-1.136	9	-1.176,-1.101	9,9	50/50	A
137	L	LEU137:	-0.359	6	-0.510,-0.172	7,6	50/50	I,L,V
138	A	ALA138:	1.862	1	0.912, 2.321	1,1	45/50	A,D,E,G,K,M,N,R,S
139	R	ARG139:	-0.953	9	-1.049,-0.919	9,9	45/50	K,R
140	I	ILE140:	-0.713	8	-0.838,-0.637	8,8	45/50	I,L,V
141	A	ALA141:	0.281	4	0.068, 0.396	5,3	45/50	A,E,H,K,L,N,Q,R,S,V
142	M	MET142:	0.548	3*	0.068, 0.912	5,1	44/50	D,E,F,M,Q,W
143	K	LYS143:	0.316	4	0.068, 0.396	5,3	44/50	D,E,K,L,M,Q,T,V,Y
144	E	GLU144:	0.491	3*	0.068, 0.912	5,1	44/50	A,E,H,K,L,M,N,Q,S,T
145	T	THR145:	-0.088	5	-0.358, 0.068	6,5	42/50	E,G,K,N,Q,S,T
146	K	LYS146:	2.139	1	2.321, 2.321	1,1	42/50	A,D,G,K,P,Q,R,S,V
147	G	GLY147:	2.017	1	2.321, 2.321	1,1	40/50	A,D,E,G,K,N,Q,R,S,T
148	D	ASP148:	2.158	1	2.321, 2.321	1,1	40/50	A,D,E,G,K,N,Q,R,S,T,V
149	A	ALA149:	0.663	2	0.396, 0.912	3,1	40/50	A,D,E,K,L,N,P,T

150	A	ALA150:	2.038	1	2.321, 2.321	1,1	40/50	A,C,D,E,G,I,K,N,Q,S,T,V
151	V	VAL151:	1.546	1	0.912, 2.321	1,1	40/50	A,D,E,I,K,L,N,Q,R,S,T,V
152	A	ALA152:	0.372	4*	0.068, 0.912	5,1	40/50	A,E,G,K,N,Q,R,S,T
153	P	PRO153:	1.137	1	0.912, 2.321	1,1	40/50	A,E,K,M,P,Q,T
154	P	PRO154:	0.976	1*	0.068, 2.321	5,1	3/50	E,N,P
155	S	SER155:	0.493	3*	-0.358, 0.912	6,1	3/50	Q,R,S
156	G	GLY156:	0.743	2*	-0.172, 2.321	6,1	3/50	G,L,S
157	C	CYS157:	-0.872	8	-0.989,-0.745	9,8	22/50	C,P,S
158	E	GLU158:	0.252	4	-0.172, 0.396	6,3	23/50	A,D,E,L,M
159	F	PHE159:	-0.525	7	-0.745,-0.358	8,6	38/50	F,Y
160	V	VAL160:	0.662	2	0.396, 0.912	3,1	38/50	A,C,D,G,K,L,M,P,R,S,T,V
161	S	SER161:	-1.013	9	-1.101,-0.989	9,9	38/50	P,S,T
162	V	VAL162:	-0.536	7	-0.745,-0.358	8,6	38/50	I,R,V
163	D	ASP163:	-1.123	9	-1.176,-1.101	9,9	38/50	D
164	P	PRO164:	-1.100	9	-1.176,-1.049	9,9	38/50	P
165	S	SER165:	-0.914	9	-0.989,-0.838	9,8	38/50	A,S,T
166	P	PRO166:	-0.707	8	-0.919,-0.510	9,7	38/50	G,L,P
167	A	ALA167:	-0.518	7	-0.745,-0.358	8,6	38/50	A,P,S
168	F	PHE168:	0.584	3	0.396, 0.912	3,1	34/50	A,F,L,M,T
169	S	SER169:	0.471	3*	0.068, 0.912	5,1	38/50	A,E,K,P,S,T
170	A	ALA170:	2.075	1	2.321, 2.321	1,1	37/50	A,D,E,G,K,L,M,P,Q,S,T
171	L	LEU171:	2.283	1	2.321, 2.321	1,1	32/50	A,D,E,F,G,H,L,N,R,S,T
172	Y	TYR172:	2.147	1	2.321, 2.321	1,1	23/50	A,D,E,K,L,N,Q,S,T,Y
173	S	SER173:	2.178	1	2.321, 2.321	1,1	22/50	A,D,E,K,Q,R,S,T
174	S	SER174:	1.474	1	0.912, 2.321	1,1	17/50	A,D,E,G,K,Q,S,T
175	T	THR175:	1.050	1*	0.068, 2.321	5,1	5/50	A,G,Q,T,V
176	D	ASP176:	1.179	1*	0.396, 2.321	3,1	5/50	A,D,K,P,S
177	E	GLU177:	0.265	4*	-0.358, 0.912	6,1	4/50	D,E,R
178	P	PRO178:	0.971	1*	0.068, 2.321	5,1	4/50	G,K,P
179	V	VAL179:	-0.140	6*	-0.838, 0.068	8,5	3/50	E,V
180	P	PRO180:	0.987	1*	0.068, 2.321	5,1	3/50	E,P,R
181	H	HIS181:	1.773	1	0.912, 2.321	1,1	38/50	A,G,H,K,L,P,Q,R,S,T,V,Y
182	T	THR182:	0.798	2	0.396, 0.912	3,1	38/50	D,E,H,K,P,S,T
183	V	VAL183:	-0.397	7	-0.637,-0.172	8,6	38/50	F,I,L,R,V
184	E	GLU184:	1.938	1	0.912, 2.321	1,1	38/50	A,D,E,G,H,P,Q,S,T
185	E	GLU185:	0.950	1	0.396, 0.912	3,1	38/50	A,D,E,K,L,Q,R,S,T
186	L	LEU186:	-0.676	8	-0.838,-0.510	8,7	38/50	A,F,I,L
187	E	GLU187:	0.315	4	0.068, 0.396	5,3	38/50	A,E,G,K,Q,R
188	A	ALA188:	1.856	1	0.912, 2.321	1,1	38/50	A,D,E,K,N,Q,R,S,T
189	V	VAL189:	1.712	1	0.912, 2.321	1,1	38/50	A,D,E,I,K,L,N,Q,R,T,V
190	L	LEU190:	-0.680	8	-0.838,-0.510	8,7	38/50	F,I,L,Y

191	L	LEU191:	-0.395	7	-0.637,-0.172	8,6	38/50	I,L,M,N,V
192	D	ASP192:	-0.865	8	-0.989,-0.745	9,8	38/50	D,N
193	T	THR193:	-0.188	6	-0.358, 0.068	6,5	38/50	A,E,K,P,Q,S,T,V
194	S	SER194:	0.913	1	0.396, 0.912	3,1	38/50	A,D,E,K,N,Q,R,S,T
195	G	GLY195:	-0.371	6*	-1.049,-0.172	9,6	1/50	G
196	R	ARG196:	-0.371	6*	-0.989, 0.068	9,5	1/50	R
197	T	THR197:	1.966	1	2.321, 2.321	1,1	38/50	A,C,E,K,L,Q,R,T
198	R	ARG198:	0.283	4	0.068, 0.396	5,3	38/50	C,D,G,P,R,S,T
199	L	LEU199:	-0.854	8	-0.989,-0.745	9,8	38/50	I,L,M
200	F	PHE200:	-0.822	8	-0.989,-0.745	9,8	38/50	F,Y
201	R	ARG201:	1.540	1	0.912, 2.321	1,1	38/50	D,E,H,K,L,N,Q,R,S,Y
202	R	ARG202:	-1.122	9	-1.176,-1.101	9,9	38/50	R
203	Y	TYR203:	-1.084	9	-1.176,-1.049	9,9	38/50	Y
204	M	MET204:	-0.839	8	-0.989,-0.745	9,8	38/50	A,M,Q,R
205	A	ALA205:	-1.063	9	-1.144,-0.989	9,9	38/50	A,V
206	M	MET206:	-1.046	9	-1.144,-0.989	9,9	38/50	L,M
207	F	PHE207:	-1.089	9	-1.176,-1.049	9,9	38/50	F
208	T	THR208:	-0.519	7	-0.745,-0.358	8,6	38/50	A,G,R,S,T,Y
209	L	LEU209:	-1.092	9	-1.176,-1.049	9,9	38/50	L
210	R	ARG210:	-1.122	9	-1.176,-1.101	9,9	38/50	R
211	N	ASN211:	-0.639	8	-0.745,-0.510	8,7	38/50	D,N
212	L	LEU212:	0.314	4	0.068, 0.396	5,3	38/50	A,D,F,H,I,L,M,V
213	A	ALA213:	0.046	5	-0.172, 0.396	6,3	38/50	A,C,G,K,R,S
214	T	THR214:	-0.109	5	-0.358, 0.068	6,5	38/50	A,D,G,H,N,R,S,T
215	E	GLU215:	0.246	4	-0.172, 0.396	6,3	38/50	D,E,G,K,P,R,S
216	A	ALA216:	0.366	4*	0.068, 0.912	5,1	38/50	A,D,E,H,K,P,Q,R
217	A	ALA217:	-0.915	9	-0.989,-0.838	9,8	38/50	A,S
218	V	VAL218:	-0.474	7	-0.637,-0.358	8,6	38/50	A,C,I,V
219	A	ALA219:	0.772	2	0.396, 0.912	3,1	38/50	A,D,E,H,K,L,Q,S,Y
220	A	ALA220:	-1.064	9	-1.144,-1.049	9,9	38/50	A,S
221	L	LEU221:	-0.849	8	-0.989,-0.745	9,8	38/50	I,L
222	C	CYS222:	-0.619	7	-0.745,-0.510	8,7	38/50	A,C,G,T,V
223	R	ARG223:	0.576	3	0.396, 0.912	3,1	38/50	A,D,E,K,Q,R,S,T
224	G	GLY224:	-0.719	8	-0.919,-0.637	9,8	38/50	A,G,S
225	L	LEU225:	0.066	5	-0.172, 0.396	6,3	38/50	F,L,M
226	R	ARG226:	2.185	1	2.321, 2.321	1,1	16/50	D,E,G,H,K,Q,R,S,Y
227	E	GLU227:	2.182	1	2.321, 2.321	1,1	38/50	A,C,D,E,G,I,K,N,Q,R,S,V
228	D	ASP228:	-0.446	7	-0.637,-0.358	8,6	35/50	A,D,E,G,K,Q
229	N	ASN229:	0.068	5	-0.172, 0.396	6,3	29/50	D,E,G,N,P,R,S
230	V	VAL230:	-0.371	6*	-0.989, 0.068	9,5	1/50	V
231	S	SER231:	-1.080	9	-1.144,-1.049	9,9	38/50	G,S

232	A	ALA232:	-0.912	9	-0.989,-0.838	9,8	38/50	A,P,S
233	L	LEU233:	-1.092	9	-1.176,-1.049	9,9	38/50	L
234	F	PHE234:	-0.950	9	-1.101,-0.838	9,8	38/50	F,L
235	R	ARG235:	-0.760	8	-0.919,-0.637	9,8	38/50	K,R
236	H	HIS236:	-1.128	9	-1.176,-1.101	9,9	38/50	H
237	E	GLU237:	-1.117	9	-1.176,-1.101	9,9	38/50	E
238	V	VAL238:	-0.548	7	-0.745,-0.358	8,6	38/50	I,V
239	A	ALA239:	-0.888	9	-0.989,-0.838	9,8	38/50	A,C,G,S
240	F	PHE240:	-0.462	7	-0.745,-0.358	8,6	38/50	F,Y
241	V	VAL241:	-1.062	9	-1.144,-0.989	9,9	38/50	I,V
242	L	LEU242:	-0.827	8	-0.989,-0.745	9,8	38/50	F,L
243	G	GLY243:	-1.090	9	-1.176,-1.049	9,9	38/50	G
244	Q	GLN244:	-1.041	9	-1.101,-0.989	9,9	38/50	E,Q
245	L	LEU245:	-0.021	5	-0.358, 0.068	6,5	38/50	I,L,M
246	E	GLU246:	0.239	4	0.068, 0.396	5,3	38/50	A,C,E,G,I,L,Q,R,S
247	R	ARG247:	0.128	4	-0.172, 0.396	6,3	38/50	A,D,E,H,N,R,S
248	P	PRO248:	-0.360	6	-0.637,-0.172	8,6	38/50	E,K,P,R,T
249	S	SER249:	-0.107	5	-0.358, 0.068	6,5	38/50	A,C,E,H,S,T,V,Y
250	S	SER250:	-0.587	7	-0.745,-0.510	8,7	38/50	A,C,S,T,V
251	Q	GLN251:	-0.250	6	-0.510,-0.172	7,6	38/50	I,L,Q,S,T,V
252	P	PRO252:	-0.593	7	-0.838,-0.510	8,7	38/50	A,D,P,Q,S
253	A	ALA253:	0.814	2	0.396, 0.912	3,1	38/50	A,C,E,F,G,H,P,Q,S,T,Y
254	L	LEU254:	-0.969	9	-1.101,-0.919	9,9	38/50	L,M
255	I	ILE255:	0.316	4	0.068, 0.396	5,3	38/50	A,I,K,L,Q,S,T,V
256	A	ALA256:	0.342	4*	0.068, 0.912	5,1	38/50	A,D,E,H,K,S,T
257	A	ALA257:	0.201	4	-0.172, 0.396	6,3	38/50	A,C,G,N,R,T,V
258	L	LEU258:	-0.837	8	-0.989,-0.745	9,8	38/50	L,M,V
259	K	LYS259:	0.966	1	0.396, 0.912	3,1	38/50	A,E,G,K,L,N,Q,R,S
260	D	ASP260:	0.447	3*	0.068, 0.912	5,1	38/50	D,K,L,N,Q,R,S
261	E	GLU261:	2.127	1	2.321, 2.321	1,1	38/50	A,C,E,F,H,K,L,M,P,Q,R,S,T,V
262	E	GLU262:	2.262	1	2.321, 2.321	1,1	38/50	A,D,E,H,K,L,N,P,R,S,T
263	E	GLU263:	-1.117	9	-1.176,-1.101	9,9	38/50	E
264	A	ALA264:	0.164	4	-0.172, 0.396	6,3	38/50	A,D,E,H,M,N,S,V
265	P	PRO265:	0.619	3	0.396, 0.912	3,1	38/50	A,C,D,E,G,P,S
266	M	MET266:	-1.130	9	-1.176,-1.101	9,9	38/50	M
267	V	VAL267:	-1.132	9	-1.176,-1.101	9,9	38/50	V
268	R	ARG268:	-1.122	9	-1.176,-1.101	9,9	38/50	R
269	H	HIS269:	-1.128	9	-1.176,-1.101	9,9	38/50	H
270	E	GLU270:	-1.117	9	-1.176,-1.101	9,9	38/50	E
271	A	ALA271:	-0.953	9	-1.049,-0.919	9,9	38/50	A,C
272	A	ALA272:	-1.133	9	-1.176,-1.101	9,9	38/50	A

273	E	GLU273:	-1.117	9	-1.176,-1.101	9,9	38/50	E
274	A	ALA274:	-1.133	9	-1.176,-1.101	9,9	38/50	A
275	L	LEU275:	-1.092	9	-1.176,-1.049	9,9	38/50	L
276	G	GLY276:	-1.090	9	-1.176,-1.049	9,9	38/50	G
277	A	ALA277:	-0.480	7	-0.637,-0.358	8,6	38/50	A,G,S
278	I	ILE278:	-1.055	9	-1.144,-0.989	9,9	38/50	I,L
279	A	ALA279:	-0.794	8	-0.919,-0.637	9,8	38/50	A,G,V
280	D	ASP280:	0.252	4	0.068, 0.396	5,3	38/50	A,D,E,H,K,N,R,S,T
281	P	PRO281:	1.064	1	0.396, 0.912	3,1	38/50	C,D,E,K,N,P,Q,T,V,Y
282	A	ALA282:	0.520	3*	0.068, 0.912	5,1	38/50	A,D,E,P,Q,S
283	T	THR283:	0.363	4*	0.068, 0.912	5,1	38/50	A,C,G,R,S,T,V
284	L	LEU284:	-0.215	6	-0.510, 0.068	7,5	38/50	I,L,T,V
285	P	PRO285:	0.797	2	0.396, 0.912	3,1	38/50	A,D,E,K,P,Q
286	V	VAL286:	-0.031	5	-0.358, 0.068	6,5	38/50	A,I,L,P,T,V
287	L	LEU287:	-1.092	9	-1.176,-1.049	9,9	38/50	L
288	E	GLU288:	0.578	3	0.396, 0.912	3,1	38/50	E,K,L,N,Q,R,S,T
289	S	SER289:	0.461	3*	0.068, 0.912	5,1	38/50	A,E,K,Q,R,S
290	Y	TYR290:	0.476	3*	0.068, 0.912	5,1	38/50	F,H,L,W,Y
291	A	ALA291:	0.882	2	0.396, 0.912	3,1	38/50	A,I,L,R,S,V
292	T	THR292:	1.640	1	0.912, 2.321	1,1	38/50	A,D,E,G,H,K,N,Q,T,V
293	H	HIS293:	-0.837	8	-0.989,-0.745	9,8	38/50	D,H,K,R
294	H	HIS294:	2.216	1	2.321, 2.321	1,1	38/50	A,D,E,G,H,K,M,P,Q,S
295	E	GLU295:	-0.289	6	-0.510,-0.172	7,6	38/50	C,D,E,K,V
296	P	PRO296:	1.242	1	0.912, 2.321	1,1	38/50	A,D,E,K,P,Q,R,S,T,V
297	I	ILE297:	-0.956	9	-1.049,-0.919	9,9	38/50	I,V
298	V	VAL298:	-1.058	9	-1.144,-0.989	9,9	38/50	L,V
299	R	ARG299:	-0.663	8	-0.838,-0.510	8,7	38/50	A,K,R,S
300	D	ASP300:	-0.734	8	-0.919,-0.637	9,8	38/50	D,E,Q
301	S	SER301:	-1.140	9	-1.176,-1.144	9,9	38/50	S
302	C	CYS302:	-0.219	6	-0.510, 0.068	7,5	38/50	A,C,I,V
303	V	VAL303:	-0.218	6	-0.510, 0.068	7,5	38/50	A,E,I,Q,V
304	V	VAL304:	-0.988	9	-1.101,-0.919	9,9	38/50	I,V
305	A	ALA305:	-1.133	9	-1.176,-1.101	9,9	38/50	A
306	L	LEU306:	-0.832	8	-0.989,-0.745	9,8	38/50	I,L,V
307	E	GLU307:	-0.947	9	-1.049,-0.838	9,8	38/50	D,E,S
308	M	MET308:	-0.715	8	-0.838,-0.637	8,8	38/50	E,I,L,M,V
309	H	HIS309:	0.932	1	0.396, 0.912	3,1	38/50	A,C,H,I,L,T,W,Y
310	K	LYS310:	-0.467	7	-0.637,-0.358	8,6	38/50	A,D,E,K,S
311	Y	TYR311:	-0.242	6	-0.510, 0.068	7,5	38/50	F,H,Y
312	W	TRP312:	0.594	3*	-0.172, 0.912	6,1	9/50	E,F,W
313	A	ALA313:	0.026	5*	-0.510, 0.396	7,3	9/50	A,K,N

314	N	ASN314:	-1.002	9	-1.144,-0.919	9,9	9/50	N
315	F	PHE315:	-0.371	6*	-0.989, 0.068	9,5	1/50	F
316	N	ASN316:	-0.371	6*	-1.049, 0.068	9,5	1/50	N
317	G	GLY317:	-0.371	6*	-1.049,-0.172	9,6	1/50	G
318	L	LEU318:	-0.371	6*	-0.989, 0.068	9,5	1/50	L
319	A	ALA319:	-0.371	6*	-1.049,-0.172	9,6	1/50	A
320	H	HIS320:	-0.371	6*	-1.049, 0.068	9,5	1/50	H
321	Q	GLN321:	-0.371	6*	-1.049, 0.068	9,5	1/50	Q
322	Q	GLN322:	-0.371	6*	-1.049, 0.068	9,5	1/50	Q
323	Q	GLN323:	-0.371	6*	-1.049, 0.068	9,5	1/50	Q
324	Q	GLN324:	-0.371	6*	-1.049, 0.068	9,5	1/50	Q
325	E	GLU325:	-0.371	6*	-1.049, 0.068	9,5	1/50	E
326	A	ALA326:	-0.371	6*	-1.049,-0.172	9,6	1/50	A

*Below the confidence cut-off - The calculations for this site were performed on less than 6 non-gaped homologue sequences, or the confidence interval for the estimated score is equal to- or larger than- 4 color grades.
